# Supplementary material for: Shank Proteins Couple the Endocytic Zone to the Postsynaptic Density to Control Trafficking and Signaling of Metabotropic Glutamate Receptor 5
Source: Cell Rep. 2019 Oct 8;29(2):258–269.e8. doi: 10.1016/j.celrep.2019.08.102 (PMC6815225; doi:10.1016/j.celrep.2019.08.102)
Supplement: Document S1. Figures S1–S4 and Tables S1 and S2 [file mmc1.pdf]

Cell Reports, Volume *29*

Supplemental Information

Shank Proteins Couple the Endocytic Zone to the Postsynaptic Density to Control Trafficking and Signaling of Metabotropic Glutamate Receptor 5

Nicky Scheefhals, Lisa A.E. Catsburg, Margriet L. Westerveld, Thomas A. Blanpied, Casper C. Hoogenraad, and Harold D. MacGillavry

Supplementary Figure 1

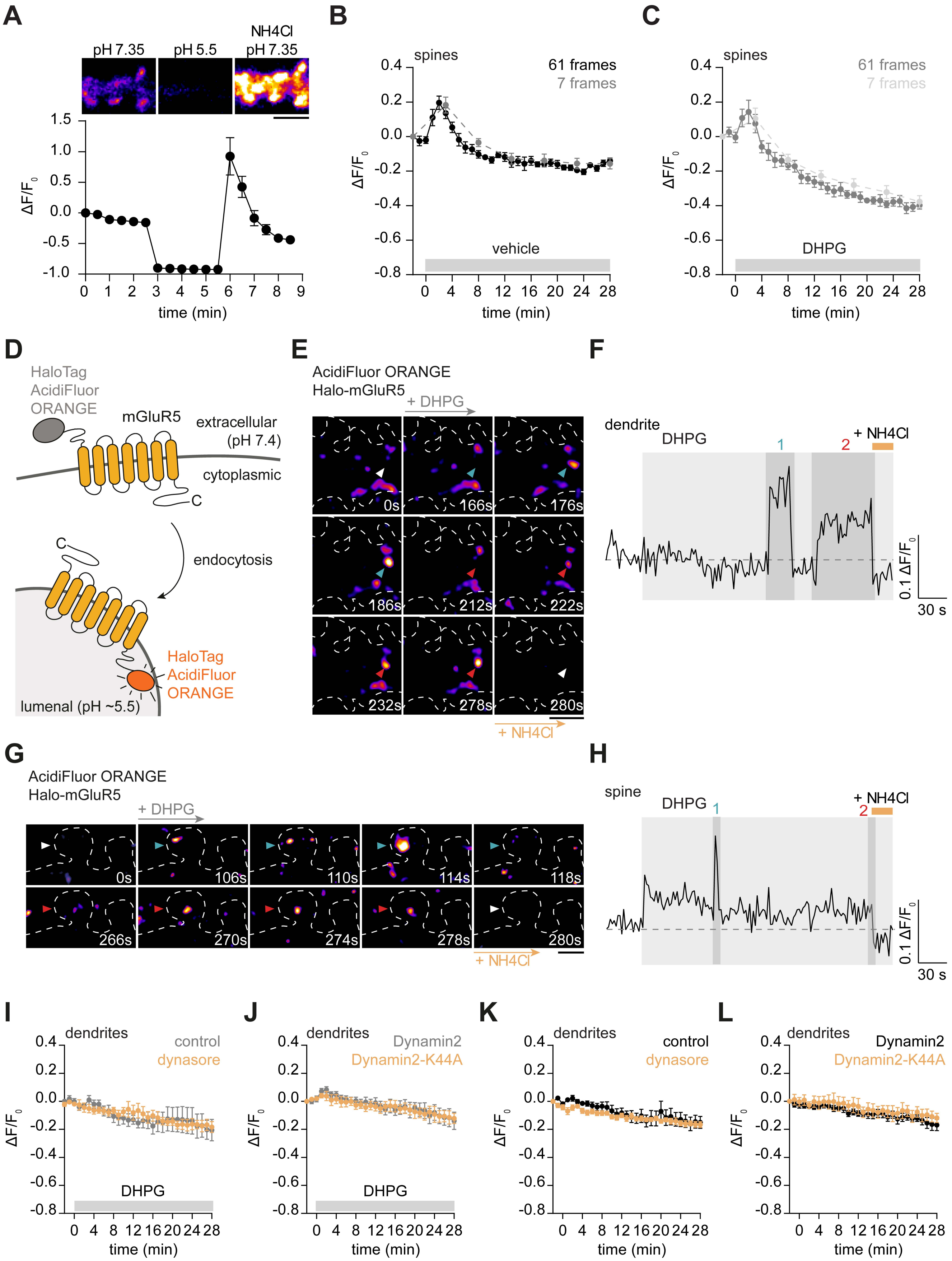

Figure S1, related to Figure 1. Characterization of SEP-mGluR5 fluorescence and evaluation of dendritic internalization

(A) Surface SEP-mGluR5 fluorescence intensity in imaging buffer with pH 7.35 (t = 0 - 3 min), is quenched at pH 5.5 (t = 3 – 6 min) and increased in fluorescence upon the application of imaging buffer containing NH_4Cl with pH 7.35 (t = 6 min) visualizing both surface and intracellular SEP-mGluR5 (n = 6). Scale bar, 2 µm.

(B and C) Quantification of SEP-mGluR5 intensity in spines over a 30-minute time period comparing the loss of SEP-mGluR5 intensity when imaged every 5 minutes (7 frames; dashed light grey line; n = 12) and when imaged every 30 seconds (61 frames; solid black line; n = 8) after application with vehicle (B) and after DHPG stimulation (7 frames; dashed light grey line; n = 12 and 61 frames; solid dark grey line; n= 6) (C). The data sets from 61 frames shown in B and C are also shown in Figure 1G, as these figures describe different aspects of the same experiment.

(D) Schematic of Halo-tag labeled with AcidiFluor ORANGE fused to mGluR5 to reveal acidification of Halo-mGluR5-containing endocytic vesicles.

(E and G) Representative image of a dendrite expressing Halo-mGluR5 labeled with AcidiFluor ORANGE stimulated with DHPG (at t = 40 s) showing two acidification events, and quenching of the signal upon application of imaging buffer containing NH_4Cl with pH 7.35 (t = 280 s) at the dendritic shaft (E) and in a spine (G). Arrowheads indicate two acidification events (1; blue and 2; red). Scale bars, 2 µm.

(F and H) $\Delta F/F_0$ trace of the Halo-mGluR5 signal intensity, showing the baseline (t = 0 – 40 s), application of DHPG (t = 42 – 178 s; light grey), the acidification events shown in E and G (dark grey) and the application of NH_4Cl (t = 280 – 300 s; orange) at the dendritic shaft shown in E (indicated by arrowhead) (F) and in the spine shown in G (indicated by arrowhead) (H).

(I and J) Quantification of SEP-mGluR5 intensity in dendrites over time after DHPG stimulation comparing the time course of SEP-mGluR5 intensity in control neurons (grey; n = 6) with neurons pre-treated with dynasore (orange; n = 6) (I) and in neurons co-transfected with Dyn2 (grey; n = 6) with neurons co-transfected with the dominant negative Dyn2-K44A (orange; n = 6) (J).

(K and L) Quantification of SEP-mGluR5 intensity in dendrites over time without the addition of DHPG comparing the time course of SEP-mGluR5 intensity in control neurons (black; n = 6) with neurons pre-treated with dynasore (orange; n = 8) (K) and in neurons co-transfected with Dyn2 (grey; n = 6) with neurons co-transfected with the dominant negative Dyn2-K44A (orange; n = 6) (L).

Data are represented as mean ± SEM.

Supplementary Figure 2

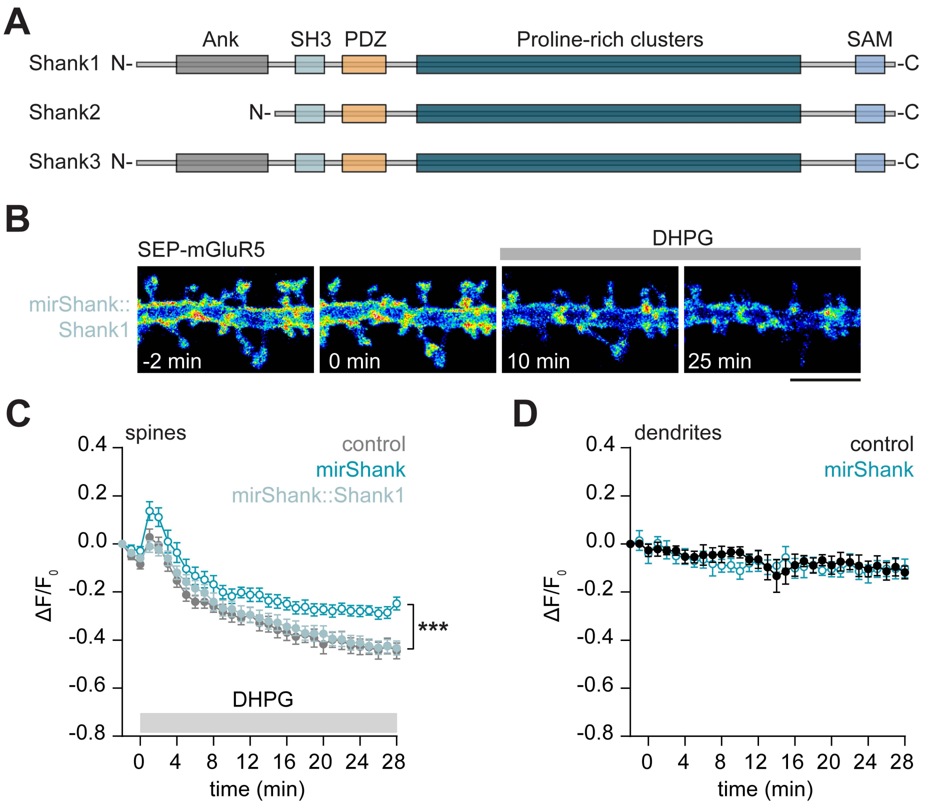

Figure S2, related to Figure 2. Re-expression of Shank1 rescues agonist-induced mGluR5 internalization in spines

(A) Domain structure of Shank1, Shank2 and Shank3.

(B) Representative live-cell time-lapse image of SEP-mGluR5 before and after DHPG stimulation (added at t = 0 min) in mirShank::Shank1 neurons. Scale bar, 5 µm.

(C) Quantification of SEP-mGluR5 intensity in spines over time after the addition of DHPG comparing the time course of SEP-mGluR5 intensity in control (grey; n = 14), mirShank (blue; open circles; n = 17) and mirShank::Shank1 rescue neurons (shade of blue; closed circles; n = 22).

(D) Quantification of SEP-mGluR5 intensity in dendrites over time without the addition of DHPG comparing the time course of SEP-mGluR5 intensity in control (black; n = 5) and mirShank neurons (blue; n = 5).

Data are represented as mean ± SEM. ***, indicates $P < 0.001$.

Supplementary Figure 3

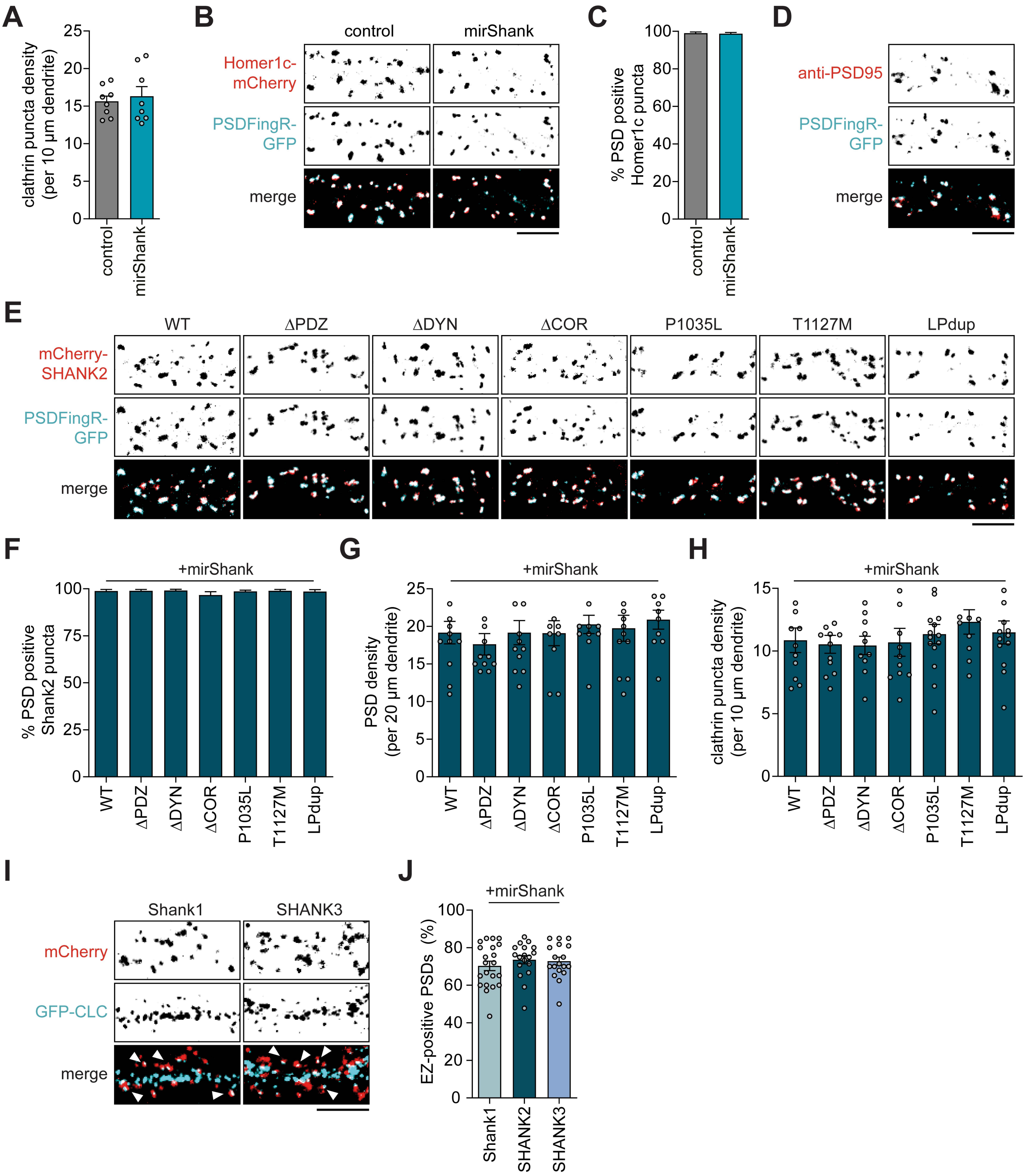

Figure S3, related to Figure 3. Clathrin puncta density in dendrites, mGluR5 spine enrichment and synaptic targeting of SHANK2 mutants

(A) Quantification of the density of GFP-CLC puncta along the dendrite (per 10 µm) in control (n = 8) and mirShank (n=8) neurons.

(B) Representative images of dendrites co-expressing PSDFingR-GFP (cyan) and Homer1c-mCherry (red) in control and mirShank neurons. Scale bar, 5 µm.

(C) Quantification of the percentage of Homer1c-mCherry puncta positive for PSDFingR-GFP, a marker of the PSD, in control (n = 10) and mirShank (n = 11) neurons.

(D) Co-localization of PSDFingR-GFP (cyan) and immuno-labeled anti-PSD-95 (red). Scale bar, 5 µm.

(E) Representative images of dendrites co-expressing mCherry-tagged SHANK2 rescue constructs (red) and PSDFingR-GFP (cyan). Scale bar, 5 µm.

(F) Quantification of the percentage of mCherry-tagged WT and mutant SHANK2 puncta positive for PSDFingR-GFP, a marker of the PSD (WT: n = 12, Δ PDZ: n = 11, ΔDYN: n = 12, ΔCOR: n = 10, P1035L: n = 11, T1127M: n = 12, LPdup: n = 10).

(G) Quantification of the density of PSDs, marked by PSDFingR-GFP, along the dendrite (per 20 µm) in neurons co-expressing the mCherry-tagged SHANK2 rescue constructs (WT: n = 12, Δ PDZ: n = 11, ΔDYN: n = 12, ΔCOR: n = 10, P1035L: n = 11, T1127M: n = 12, LPdup: n = 10).

(H) Quantification of the density of GFP-CLC puncta along the dendrite (per 10 µm) in neurons co-expressing mCherry-tagged SHANK2 rescue constructs (WT: n = 11, Δ PDZ: n = 12, ΔDYN: n = 10, ΔCOR: n = 11, P1035L: n = 15, T1127M: n = 10, LPdup: n = 13).

(I) Representative images of dendrites co-expressing mCherry-tagged Shank1, SHANK2 and SHANK3 rescue constructs (red) and GFP-CLC (cyan). Scale bar, 5 µm.

(J) Quantification of the percentage of EZ-positive PSDs in neurons co-expressing mCherry-tagged Shank1 (n = 21), SHANK2 (n = 18) and SHANK3 (n = 18) rescue constructs.

Data are represented as mean ± SEM.

Supplementary Figure 4

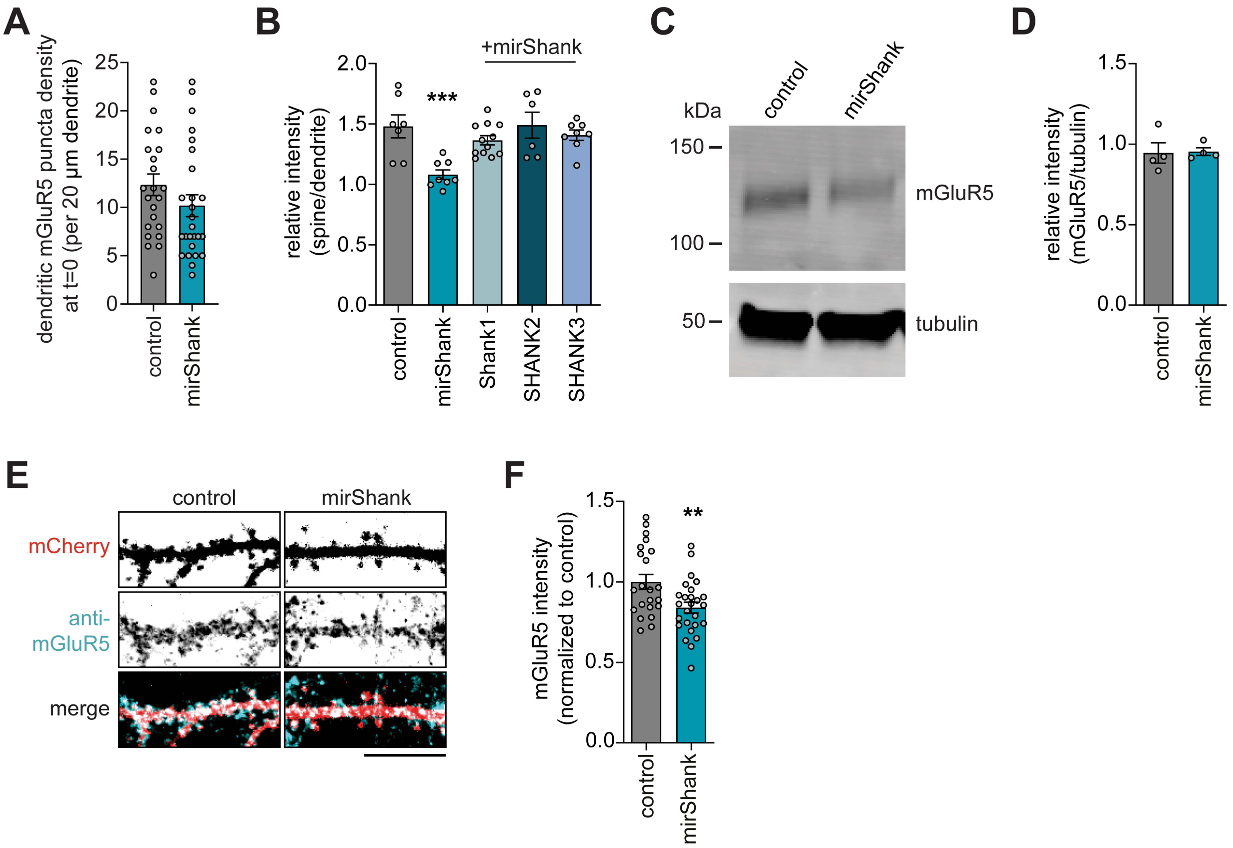

Figure S4, related to Figure 4. Shank proteins control surface and total expression of mGluR5

(A) Quantification of internalized myc-mGluR5 puncta density in dendrites of control (n = 23) and mirShank neurons (n = 26) at t = 0.
(B) Quantification of SEP-mGluR5 intensity in spines over dendritic shaft of control (n = 7), mirShank (n = 8), mirShank::Shank1 (n = 12), mirShank::SHANK2 (n = 6) and mirShank::SHANK3 (n = 9) rescue neurons.
(C) Western blot analysis of total lysates of neurons infected with GFP and GFP::mirShank and immuno-labelled for anti-mGluR5; tubulin was used as a loading control.
(D) Quantification of anti-mGluR5 over tubulin intensity in control and mirShank neurons (n = 3).
(E) Representative images of dendrites immuno-labeled for anti-mGluR5 (cyan) in mCherry-tagged control and mirShank neurons (red). Scale bar, 10 µm.
(F) Quantification of anti-mGluR5 intensity along the dendrite (20 µm) in control (n = 20) and mirShank (n = 27) neurons, normalized to the average intensity of anti-mGluR5 fluorescence in the control condition.
Data are represented as mean ± SEM. **, indicates $P < 0.01$ and ***, indicates $P < 0.001$.

Table S1, related to STAR Methods. miRNA targeting sequences

Targeting protein	miRNA sequence	Source
Shank1	ACAGACCAACCTGGATGAGAA	(MacGillavry et al., 2015)
Shank2	GGACTTGGATGAGGACTTTCT	(MacGillavry et al., 2015)
Shank3	GGAAGTCACCAGAGGACAAGA	(MacGillavry et al., 2015)

Table S2, related to STAR Methods. Statistical Analysis

Figure	Test	Condition	*P* value condition		*P value condition x time*
**1D**	ANOVA	ALL	<0.0001	***	
	ANOVA with TUKEY	EEA1 vs. Rab5	0.3715	ns	
		EEA1 vs. Rab11	0.9063	ns	
		EEA1 vs. TfR	0.8299	ns	
		EEA1 vs. Rab7	<0.0001	***	
		EEA1 vs. LAMP1	<0.0001	***	
		Rab5 vs. Rab11	0.9599	ns	
		Rab5 vs. TfR	0.9723	ns	
		Rab5 vs. Rab7	<0.0001	***	
		Rab5 vs. LAMP1	<0.0001	***	
		Rab11 vs. TfR	>0.9999	ns	
		Rab11 vs. Rab7	<0.0001	***	
		Rab11 vs. LAMP1	<0.0001	***	
		TfR vs. Rab7	<0.0001	***	
		TfR vs. LAMP1	<0.0001	***	
		Rab7 vs. LAMP1	0.6920	ns	
**1G**	2-way RM ANOVA	vehicle vs. DHPG	0.0008	***	<0.0001
**1H**	2-way RM ANOVA	vehicle vs. DHPG	0.1363	ns	0.1082
**1I**	2-way RM ANOVA	control vs. dynasore	0.0136	*	0.0001
**1J**	2-way RM ANOVA	control vs. Dyn2-K44A	0.0136	*	<0.0001
**1K**	2-way RM ANOVA	control vs. dynasore	0.6672	ns	<0.0001
**1L**	2-way RM ANOVA	control vs. Dyn2-K44A	0.9663	ns	0.9993
**S1B**	2-way RM ANOVA for 7 matching time points	61 frames vs. 7 frames	0.4483	ns	0.4409
**S1C**	2-way RM ANOVA for 7 matching time points	61 frames vs. 7 frames	0.3799	ns	0.9534
**S1I**	2-way RM ANOVA	control vs. dynasore	0.8814	ns	0.2119
**S1J**	2-way RM ANOVA	control vs. Dyn2-K44A	0.8080	ns	>0.9999
**S1K**	2-way RM ANOVA	control vs. dynasore	0.4029	ns	0.9983
**S1L**	2-way RM ANOVA	control vs. Dyn2-K44A	0.4755	ns	0.9829
**2B**	2-way RM ANOVA	control vs. mirShank	<0.0001	***	<0.0001
**2C**	2-way RM ANOVA	control vs. mirShank	0.5636	ns	0.0004
**2D**	2-way RM ANOVA	ALL	0.0026	**	<0.0001

	2-way RM ANOVA with Tukey	mirShank vs. Control<br>mirShank::SHANK2 vs. Control<br>mirShank::SHANK3 vs. Control<br>mirShank::SHANK2 vs. mirShank<br>mirShank::SHANK3 vs. mirShank<br>mirShank::SHANK3 vs. mirShank::SHANK2	<0.0001<br>0.9997<br>0.4561<br><0.0001<br><0.0001<br>0.5395	***<br>ns<br>ns<br>***<br>***<br>ns	
**2E**	2-way RM ANOVA	control vs. SHANK2 OE	0.4699	ns	<0.0001
**2F**	2-way RM ANOVA	control vs. mirShank	0.7244	ns	0.6593
**2G**	Mixed-effects ANOVA	control vs. mirShank	0.0001	***	<0.0001
**S2C**	2-way RM ANOVA	ALL	0.0082	**	<0.0001
	2-way RM ANOVA with Tukey	control vs. mirShank<br>control vs. mirShank::SHANK1<br>mirShank vs. mirShank::SHANK1	<0.0001<br>0.3235<br><0.0001	***<br>ns<br>***	
**S2D**	2-way RM ANOVA	control vs. mirShank	0.7976	ns	0.2373
**3C**	Unpaired t-test	control vs. mirShank	<0.0001	***	
**3D**	ANOVA	ALL	<0.0001	***	
	ANOVA with Tukey	untransfected vs. control<br>untransfected vs. mirShank<br>control vs. mirShank	0.5145<br>0.0001<br><0.0001	ns<br>***<br>***	
**3F**	ANOVA	ALL	<0.0001	***	
	ANOVA with Dunnet’s	WT vs. ΔPDZ<br>WT vs. ΔDYN<br>WT vs. ΔCOR<br>WT vs. P1035L<br>WT vs. T1127M<br>WT vs. LPdup	0.9998<br><0.0001<br><0.0001<br><0.0001<br><0.0001<br>0.9979	ns<br>***<br>***<br>***<br>***<br>ns	
**3H**	Unpaired t-test	control vs. mirShank	0.0012	**	
**3J**	ANOVA	ALL	<0.0001	***	
	ANOVA with Dunnet’s	WT vs. ΔPDZ<br>WT vs. ΔDYN<br>WT vs. ΔCOR<br>WT vs. P1035L<br>WT vs. T1127M<br>WT vs. LPdup	0.9999<br>0.0005<br>0.0068<br>0.0021<br>0.0003<br>0.8160	ns<br>***<br>**<br>**<br>***<br>ns	
**3L**	Paired t-test	30’: EZ+ vs. EZ-<br>60’: EZ+ vs. EZ-	0.0453<br>0.0123	*<br>*	
**S3A**	Unpaired t-test	control vs. mirShank	0.6554	ns	
**S3C**	Mann Whitney test	control vs. mirShank	0.8216	ns	
**S3F**	Kruskall Wallis	ALL	0.8980	ns	
	Kruskall Wallis with Dunn’s	WT vs. ΔPDZ<br>WT vs. ΔDYN<br>WT vs. ΔCOR<br>WT vs. P1035L<br>WT vs. T1127M<br>WT vs. LPdup	>0.9999<br>>0.9999<br>>0.9999<br>>0.9999<br>>0.9999<br>>0.9999	ns<br>ns<br>ns<br>ns<br>ns<br>ns	
**S3G**	ANOVA	ALL	0.8440	ns	
	ANOVA with Dunnet’s	WT vs. ΔPDZ	0.9523	ns	

		WT vs. ΔDYN<br>WT vs. ΔCOR<br>WT vs. P1035L<br>WT vs. T1127M<br>WT vs. LPdup	>0.9999<br>>0.9999<br>0.9903<br>0.9996<br>0.9268	ns<br>ns<br>ns<br>ns<br>ns	
**S3H**	ANOVA	ALL	0.7983	ns	
	ANOVA with Dunnet's	WT vs. ΔPDZ<br>WT vs. ΔDYN<br>WT vs. ΔCOR<br>WT vs. P1035L<br>WT vs. T1127M<br>WT vs. LPdup	0.9996<br>0.9995<br>0.9998<br>0.9963<br>0.7478<br>0.9913	ns<br>ns<br>ns<br>ns<br>ns<br>ns	
**S3J**	ANOVA	ALL	0.5627	ns	
	ANOVA with Tukey	Shank1 vs. SHANK2<br>Shank1 vs. SHANK3<br>SHANK2 vs. SHANK3	0.5679<br>0.7157<br>0.9715	ns<br>ns<br>ns	
**4B**	2-way ANOVA	control vs. mirShank	0.2719	ns	0.0129
	2-way ANOVA with Tukey	0':control vs. 2':control<br>0':control vs. 5':control<br>0':control vs. 10':control<br>0':control vs. 20':control<br>0':mirShank vs. 2':mirShank<br>0':mirShank vs. 5':mirShank<br>0':mirShank vs. 10':mirShank<br>0':mirShank vs. 20':mirShank<br>2':control vs. 2':mirShank<br>5':control vs. 5':mirShank<br>10':control vs. 10':mirShank<br>20':control vs. 20':mirShank	>0.9999<br>0.4864<br>0.9977<br>0.6553<br>>0.9999<br>0.9301<br>0.0002<br>0.6414<br>>0.9999<br>0.9997<br>0.0108<br>>0.9999	ns<br>ns<br>ns<br>ns<br>ns<br>ns<br>***<br>ns<br>ns<br>ns<br>*<br>ns	
**4C**	2-way RM ANOVA	control vs. mirShank	0.8781	ns	>0.9999
**4D**	Unpaired t-test	control vs. mirShank	0.8567	ns	
**4G**	Paired t-test	DHPG vs. DHPG + MPEP	0.0023	**	
**4J**	Mann Whitney test	control vs. mirShank	0.0043	**	
**4L**	2-way ANOVA	ALL	0.0007	***	0.1260
	2-way ANOVA with Tukey	NT:control vs. NT:mirShank<br>NT:control vs. 10':control<br>NT:control vs. 10':mirShank<br>NT:control vs. 30':control<br>NT:control vs. 30':mirShank<br>NT:mirShank vs. 10':control<br>NT:mirShank vs. 10':mirShank<br>NT:mirShank vs. 30':control<br>NT:mirShank vs. 30':mirShank<br>10':control vs. 10':mirShank<br>10':control vs. 30':control<br>10':control vs. 30':mirShank<br>10':mirShank vs. 30':control<br>10':mirShank vs. 30':mirShank	0.9997<br>0.2464<br>0.9998<br>0.0885<br>0.9889<br>0.1676<br>>0.9999<br>0.0579<br>0.9998<br>0.1217<br>0.9986<br>0.0354<br>0.0334<br>0.9992	ns<br>ns<br>ns<br>ns<br>ns<br>ns<br>ns<br>ns<br>ns<br>ns<br>ns<br>*<br>*<br>ns	

		30’:control vs. 30’:mirShank	0.0064	**	
**S4A**	Unpaired t-test	control vs. mirShank	0.1874	ns	
**S4B**	ANOVA	ALL	0.0003	***	
	ANOVA with Tukey	control vs. mirShank	0.0009	***	
		control vs. Shank1	0.6477	ns	
		control vs. SHANK2	>0.9999	ns	
		control vs. SHANK3	0.9299	ns	
		mirShank vs. Shank1	0.0094	**	
		mirShank vs. SHANK2	0.0011	**	
		mirShank vs. SHANK3	0.0059	**	
		Shank1 vs. SHANK2	0.6121	ns	
		Shank1 vs. SHANK3	0.9838	ns	
		SHANK2 vs. SHANK3	0.9029	ns	
**S4D**	Unpaired t-test	control vs. mirShank	0.9032	ns	
**S4F**	Unpaired t-test	control vs. mirShank	0.0064	**	
