# Supplementary material for: Shank Proteins Couple the Endocytic Zone to the Postsynaptic Density to Control Trafficking and Signaling of Metabotropic Glutamate Receptor 5
Source: Cell Rep. 2019 Oct 8;29(2):258–269.e8. doi: 10.1016/j.celrep.2019.08.102 (PMC6815225; doi:10.1016/j.celrep.2019.08.102)
Supplement: Document S2. Article plus Supplemental Information [file mmc2.pdf]

Cell Reports

Report

Shank Proteins Couple the Endocytic Zone to the Postsynaptic Density to Control Trafficking and Signaling of Metabotropic Glutamate Receptor 5

Graphical Abstract

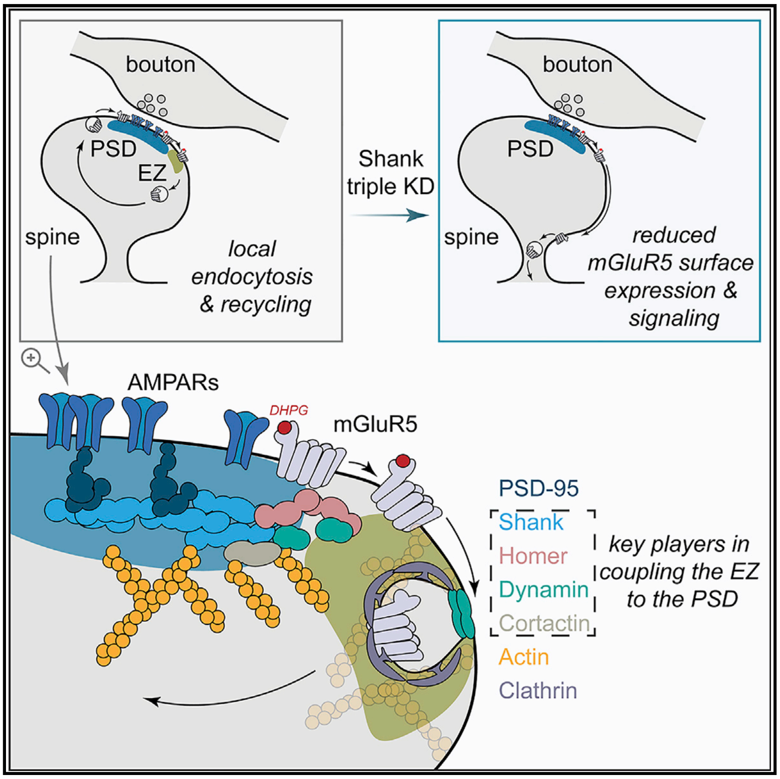

Highlights

- Receptor activation triggers efficient internalization of mGluR5 in spines
- Shank proteins control mGluR5 trafficking and signaling
- Shanks link essential components of the endocytic zone to the postsynaptic density
- Mutation in SHANK2 found in ASD disrupt these processes

Authors

Nicky Scheefhals, Lisa A.E. Catsburg, Margriet L. Westerveld, Thomas A. Blanpied, Casper C. Hoogenraad, Harold D. MacGillavry

Correspondence

h.d.macgillavry@uu.nl

In Brief

mGluR5 activity is critical for neuronal functioning, but it is unclear how membrane trafficking of mGluR5 is controlled at excitatory synapses. Scheefhals et al. show that Shank proteins anchor the endocytic machinery to the postsynaptic density to govern the efficient trafficking and signaling of mGluR5 at synapses to modulate neuronal functioning.

Scheefhals et al., 2019, Cell Reports *29*, 258–269
October 8, 2019 © 2019 The Author(s).
https://doi.org/10.1016/j.celrep.2019.08.102

CellPress

OPEN ACCESS
CellPress

Cell Reports
Report

Shank Proteins Couple the Endocytic Zone to the Postsynaptic Density to Control Trafficking and Signaling of Metabotropic Glutamate Receptor 5

Nicky Scheefhals,[1] Lisa A.E. Catsburg,[1] Margriet L. Westerveld,[1] Thomas A. Blanpied,[2] Casper C. Hoogenraad,[1] and Harold D. MacGillavry[1,3,*]
[1]Cell Biology, Neurobiology and Biophysics, Department of Biology, Faculty of Science, Utrecht University, 3584 CH Utrecht, the Netherlands
[2]Department of Physiology, Program in Neuroscience, University of Maryland School of Medicine, Baltimore, MD 21201, USA
[3]Lead Contact
*Correspondence: h.d.macgillavry@uu.nl
https://doi.org/10.1016/j.celrep.2019.08.102

SUMMARY

Activation of postsynaptic metabotropic glutamate receptors (mGluRs) modulates neuronal excitability and synaptic plasticity, while deregulation of mGluR signaling has been implicated in neurodevelopmental disorders. Overstimulation of mGluRs is restricted by the rapid endocytosis of receptors after activation. However, how membrane trafficking of mGluRs at synapses is controlled remains poorly defined. We find that in hippocampal neurons, the agonist-induced receptor internalization of synaptic mGluR5 is significantly reduced in Shank knockdown neurons. This is rescued by the re-expression of wild-type Shanks, but not by mutants unable to bind Homer1b/c, Dynamin2, or Cortactin. These effects are paralleled by a reduction in synapses associated with an endocytic zone. Moreover, a mutation in SHANK2 found in autism spectrum disorders (ASDs) similarly disrupts these processes. On the basis of these findings, we propose that synaptic Shank scaffolds anchor the endocytic machinery to govern the efficient trafficking of mGluR5 and to balance the surface expression of mGluRs to efficiently modulate neuronal functioning.

INTRODUCTION

At excitatory synapses of hippocampal neurons, the group I metabotropic glutamate receptors (mGluRs) mGluR1 and mGluR5 critically modulate synaptic transmission and plasticity (Scheefhals and MacGillavry, 2018). The contribution of mGluRs to glutamatergic signaling underlies cognitive functions, and disrupted mGluR signaling has been implicated in neurological disorders, including autism spectrum disorders (ASDs) (Lüscher and Huber, 2010). To prevent overstimulation, activated mGluRs are rapidly desensitized and internalized via clathrin-mediated endocytosis (Dhami and Ferguson, 2006). Despite the importance of controlled receptor trafficking at synapses, we know little about the mechanisms that control the endocytosis and recycling of synaptic mGluRs. The endocytosis of postsynaptic membrane proteins preferentially takes place at endocytic zones (EZs) (Rosendale et al., 2017). EZs are stable clathrin assemblies coupled to the postsynaptic density (PSD) via interactions with Homer1b/c and Dynamin3 (Blanpied et al., 2002; Lu et al., 2007; Rácz et al., 2004). Disruption of the PSD-EZ coupling reduces the synaptic population of the α-amino-3-hydroxy-5-methyl-4-isoxazolepropionic acid receptors (AMPARs), and prevents plasticity-induced receptor insertion (Petrini et al., 2009). However, whether mGluRs are locally endocytosed through EZs and recycle to the synaptic membrane remains untested.

The Shank family (Shank1, -2, and -3) is an integral part of the PSD, interacting with a multitude of synaptic proteins, as well as endocytic proteins, such as Dynamin2, Cortactin, Syndapin I, and Abp1 (Kessels et al., 2001; McNiven et al., 2000; Naisbitt et al., 1999; Okamoto et al., 2001; Qualmann et al., 2004). Moreover, abrogated mGluR signaling has been found in Shank mutant models (Bariselli et al., 2016; Kouser et al., 2013; Lee et al., 2019; Verpelli et al., 2011), but how Shank proteins control mGluR function remains unknown. We hypothesized that Shank proteins recruit components of the endocytic machinery to facilitate the local regulation of receptor internalization to control mGluR function. We found that agonist-induced internalization of mGluR5 is severely affected in Shank triple knockdown neurons and present evidence that mGluR5 is internalized through the EZ coupled to the PSD by Shank-mediated interactions. We propose that Shank proteins link the EZ to the PSD to control trafficking of synaptic membrane proteins and to balance the density of receptors at the membrane to modulate neuronal functioning.

RESULTS

Efficient Internalization and Intracellular Sorting of Activated mGluR5

To test whether the activation of mGluR5 triggers endocytosis in hippocampal neurons, we live-labeled surface-expressed myc-mGluR5, and incubated neurons with the group I-specific agonist (*S*)-3,5-dihydroxyphenylglycine (DHPG). Surface expression of mGluR5 markedly decreased over time, which was best described by a single-exponential decay function with a rate constant of 0.077 ± 0.03 min^{-1}, reaching a

 © 2019 The Author(s).
This is an open access article under the CC BY license (http://creativecommons.org/licenses/by/4.0/).

OPEN
ACCESS
CellPress

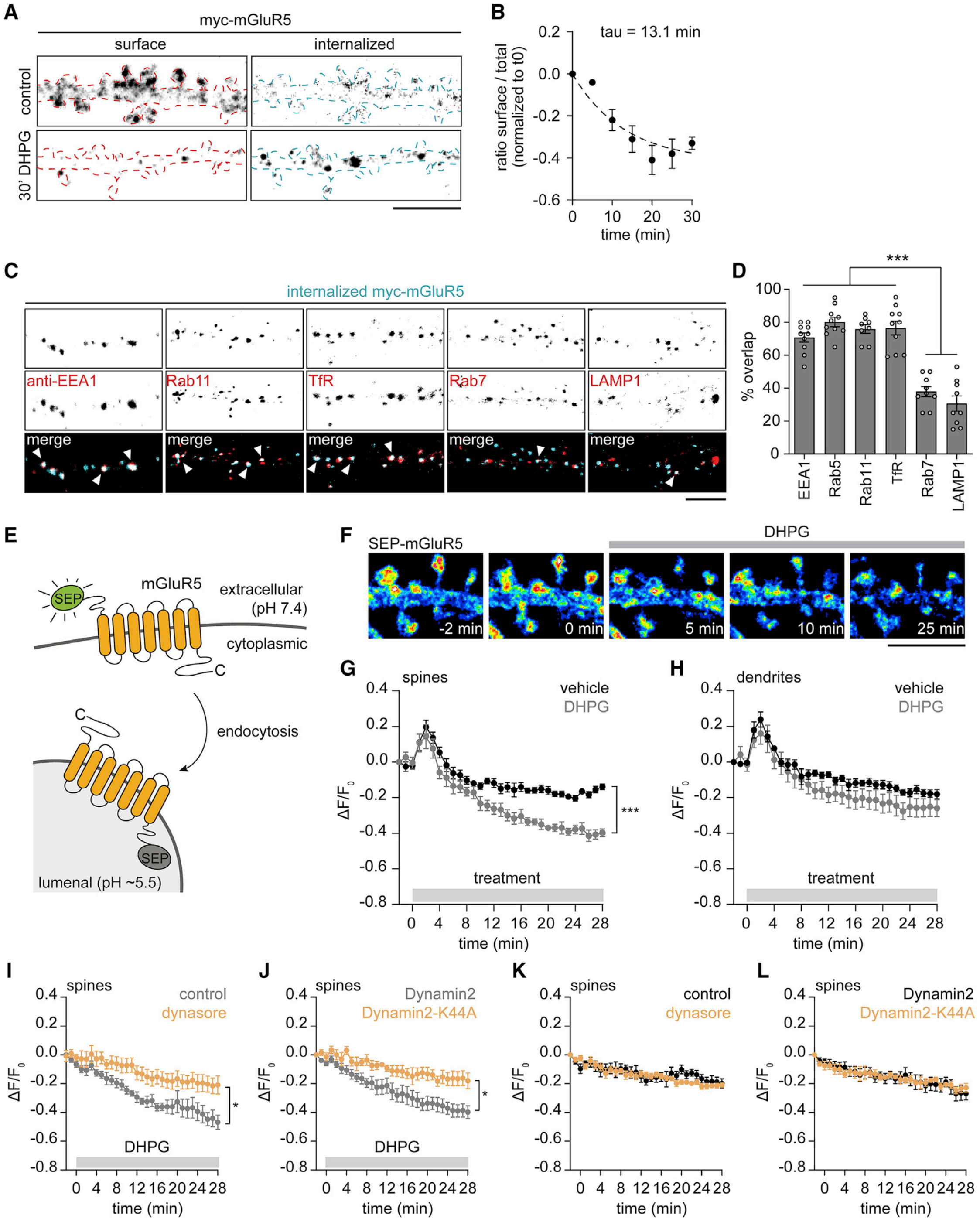

Figure 1. Efficient Agonist-Induced mGluR5 Internalization in Spines

(A) Dendrite stained for surface expressed (red outline) and internalized (cyan outline) myc-mGluR5 before (top panels) and 30 min after (bottom panels) DHPG treatment. Scale bar, 5 μm.

(B) Quantification of the ratio of surface over total myc-mGluR5 intensity at different time points after DHPG stimulation (n = 8–20). Dashed line represents single exponential fit.

(C) Co-localization of internalized myc-mGluR5 (cyan) and indicated endosomal and lysosomal markers (red). The arrowheads indicate examples of overlapping puncta. Scale bar, 5 μm.

(D) Quantification of overlap between internalized myc-mGluR5 puncta and indicated markers (EEA1: n = 10, Rab5: n = 10, Rab11: n = 8, TfR: n = 10, Rab7: n = 9, LAMP1: n = 9).

(E) Schematic of SEP-tag fused to mGluR5.

(legend continued on next page)

plateau at 42% ± 7% reduction (Figures 1A and 1B), consistent with previous reports (Lee et al., 2008). Internalized mGluR5 puncta largely overlapped with the early and recycling endosome markers anti-EEA1, GFP-Rab5, GFP-Rab11, and mRFP-TfR, but much less with the late endosome marker GFP-Rab7 and the lysosomal marker GFP-LAMP1 (EEA1: 70% ± 3%, Rab5: 80% ± 3%, Rab11: 76% ± 3%, TfR: 77% ± 4%, Rab7: 38% ± 3%, LAMP1: 31% ± 5%, $p < 0.001$; Figures 1C and 1D).

To image the surface expressed pool of mGluR5 in live cells, we tagged mGluR5 with an extracellular super-ecliptic pHluorin (SEP) tag (Figure 1E). We confirmed that the fluorescence of this GFP variant is quenched at low pH, such as in endocytic vesicles, and only becomes fluorescent at neutral pH (Figure S1A). Application of DHPG induced a rapid decrease in SEP-mGluR5 intensity from dendritic spines (DHPG: 39.7% ± 2.2% at t = 28 min, vehicle: 14.0% ± 1.6%, $p < 0.001$; Figures 1F and 1G). Imaging at reduced frame rates revealed no significant difference in observed signal reduction both after vehicle (15.8% ± 3.1%; Figure S1B) and DHPG application (37.8% ± 3.5%; Figure S1C). Thus, the observed reduction of SEP-mGluR5 intensity in unstimulated spines is not due to photobleaching, but likely reflects ongoing receptor internalization, which is consistent with other studies that estimated ~20% agonist-independent internalization over 30 min (Francesconi et al., 2009; Lee et al., 2008). In some but not all experiments, we noted that DHPG or vehicle application induced a transient increase in SEP-mGluR5 fluorescence intensity (e.g., Figure 1G). If it was observed, then it was independent of the experimental conditions and could be attributed to the opening of the imaging chamber, briefly affecting the pH of the imaging buffer.

In dendrites, the DHPG-induced decrease in SEP-mGluR5 signal was not significantly different from the vehicle control (DHPG: 26.0% ± 4.7%, vehicle: 18.1% ± 2.6%; Figure 1H). However, these measurements do not directly measure endocytosis, but also reflect ongoing recycling and lateral exchange of receptors on the membrane. To more directly determine whether mGluR5 can be internalized in dendrites, we tagged mGluR5 with an extracellular HaloTag to label with AcidiFluor ORANGE, which only fluoresces at a low pH (pH 5–6) (Isa et al., 2014) (Figure S1D). The application of DHPG induced distinct, local increases in Halo-mGluR5 signal intensity, reflecting the acidification of Halo-mGluR5 containing endocytic vesicles, both in spines and dendrites (Figures S1E–S1H).

To test whether Dynamin activity is required for agonist-induced mGluR5 internalization in spines, we treated neurons with dynasore, a potent inhibitor of Dynamin GTPase activity (Macia et al., 2006) before the addition of DHPG. Dynasore significantly reduced DHPG-induced mGluR5 internalization in spines (control: 46.9% ± 5.1%, dynasore: 20.1% ± 6.3%, $p < 0.05$; Figure 1I). Moreover, the expression of a dominant-negative form of Dynamin2 (Dyn2), Dynamin2-K44A (Dyn2-K44A), also reduced the DHPG-induced internalization of mGluR5 in spines (Dyn2: 39.8% ± 4.3%, Dyn2-K44A: 18.0% ± 5.5%, $p < 0.05$; Figure 1J). The slow decrease in the fluorescence intensity of SEP-mGluR5 observed in spines without the application of DHPG was similar in dynasore-treated neurons and neurons expressing Dyn2-K44A, and was not different from control neurons (Figures 1K and 1L). In dendrites, the decrease in SEP-mGluR5 signal, in both unstimulated and DHPG-stimulated neurons, was not affected by dynasore or the expression of Dyn2-K44A (Figures S1I–S1L), suggesting that internalization in dendrites is Dynamin-independent. These results indicate that in dendritic spines, receptor activation triggers rapid, Dynamin-dependent endocytosis of mGluR5 and that internalized receptors preferentially enter the recycling compartment.

Shank Proteins Are Required for Agonist-Induced Internalization of mGluR5 in Spines

To test whether Shank proteins contribute to mGluR5 endocytosis, we used a triple microRNA (miRNA) knockdown construct to simultaneously reduce the expression of Shank1, Shank2, and Shank3 (mirShank) (Figure S2A) (MacGillavry et al., 2016). DHPG-induced mGluR5 internalization was significantly reduced in Shank triple knockdown (hereafter, Shank knockdown) neurons compared to control neurons (control: 43.8% ± 2.2%, mirShank: 24.8% ± 2.9%, $p < 0.001$; Figures 2A and 2B). In contrast, in dendrites of both control and Shank knockdown neurons, DHPG-induced mGluR5 internalization was similar (control: 22.8% ± 2.9%, mirShank: 18.8% ± 3.5%, Figure 2C). DHPG-induced mGluR5 internalization in spines was completely restored to control levels by the re-expression of miRNA-resistant Shank1, SHANK2, or SHANK3 in Shank knockdown neurons (control: 35.3% ± 1.8%, mirShank: 10.4% ± 4.4%, mirShank::SHANK2: 36.4% ± 2.6%, mirShank::SHANK3: 36.4% ± 2.4%; Figures 2A and 2D; and control: 44.5% ± 3.3%, mirShank: 24.8% ± 2.7%, mirShank::Shank1: 43.4% ± 2.9%; Figures S2B and S2C). We did not find a significant change in agonist-induced mGluR5 internalization in neurons overexpressing SHANK2 (control: 46.6% ± 4.0%, SHANK2 overexpressing [OE]: 39.5% ± 2.7%; Figure 2E), suggesting that endogenous Shank levels are sufficient to sustain the agonist-induced endocytosis of mGluR5. Also, SEP-mGluR5 intensity was unchanged over a period of 30 min in the absence of DHPG between control and Shank knockdown neurons in spines (control: 18.6% ± 1.7%, mirShank: 22.4% ± 2.8%; Figure 2F) and dendrites (control: 11.9% ± 2.7%, mirShank: 10.1% ± 3.9%; Figure S2D). Similarly, the agonist-induced internalization of mGluR1 was also reduced in Shank knockdown neurons (control: 38.6% ± 3.4%,

(F) Live-cell time-lapse imaging of a dendrite expressing SEP-mGluR5 stimulated with DHPG at t = 0. Scale bar, 5 μm.
(G and H) Quantification of SEP-mGluR5 intensity over a 30-min period comparing the application of vehicle (black; n = 8) and DHPG (gray; n = 6) at t = 0 in spines (G) and dendrites (H).
(I and J) Quantification of SEP-mGluR5 intensity in spines over time after DHPG stimulation comparing control neurons (gray; n = 6) with neurons pre-treated with dynasore (orange; n = 6) (I) and neurons co-transfected with Dyn2 (gray; n = 6) with neurons co-transfected with Dyn2-K44A (orange; n = 6) (J).
(K and L) Quantification of SEP-mGluR5 intensity in spines over time without the addition of DHPG comparing control neurons (black; n = 6) with neurons pre-treated with dynasore (orange; n = 8) (K) and neurons co-transfected with Dyn2 (gray; n = 6) with neurons co-transfected with Dyn2-K44A (orange; n = 6) (L).
Data are represented as means ± SEMs. *$p < 0.05$, ***$p < 0.001$.

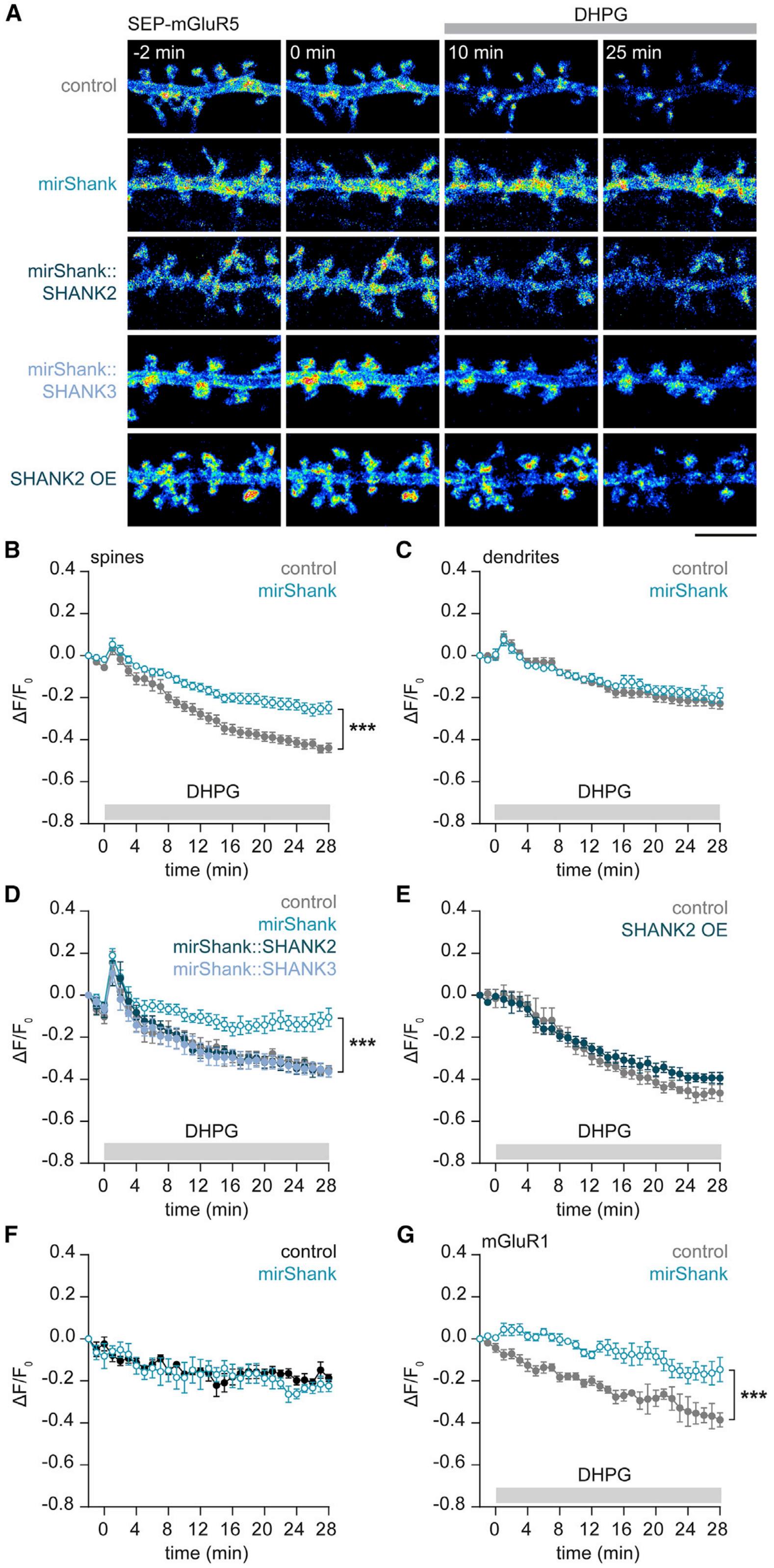

Figure 2. Shank Knockdown Reduces Agonist-Induced mGluR5 Internalization in Spines

(A) Live-cell time-lapse images of SEP-mGluR5 before and after DHPG stimulation (added at t = 0 min) in control, mirShank, mirShank::SHANK2, mirShank::SHANK3, and SHANK2 overexpression (OE) neurons. The dendrites are color-coded for the fluorescence intensity of SEP-mGluR5. Scale bar, 5 μm.

(B and C) Quantification of SEP-mGluR5 intensity over time after the addition of DHPG in spines (B) and dendrites (C) of control (gray; n = 29) and mirShank neurons (blue; n = 34).

(D and E) Quantification of SEP-mGluR5 intensity in spines over time after the addition of DHPG comparing control (gray; n = 7), mirShank (blue; open circles; n = 8), and the mirShank::SHANK2 (n = 6) and mirShank::SHANK3 (n = 8) rescue neurons (shades of blue; closed circles) (D), and comparing control (gray; n = 4) and SHANK2 overexpression (OE; blue; n = 6) neurons (E).

(F) Quantification of SEP-mGluR5 intensity in spines over time without the addition of DHPG comparing control (black; n = 5) and mirShank neurons (blue; n = 5).

(G) Quantification of SEP-mGluR1 intensity in spines over time after the addition of DHPG in control (gray; n = 8) and mirShank neurons (blue; n = 6).

Data are represented as means ± SEMs. ***$p < 0.001$.

mirShank: 14.6% ± 5.8%, $p < 0.001$; Figure 2G). Thus, agonist-induced internalization of mGluR1 and mGluR5 in spines is modulated by synaptic Shank scaffolds.

Shank Proteins Couple the EZ to the PSD to Mediate the Local Endocytosis of mGluR5

We hypothesized that Shank proteins could play a central role in positioning the EZ by recruiting essential components of the endocytic machinery to the PSD (Figure 3A). To test this, we measured the fraction of PSDs associated with an EZ in control and Shank knockdown neurons. Consistent with previous reports (Blanpied et al., 2002; Lu et al., 2007), we found that the majority of PSDs (72% ± 2%) were associated with an EZ marked by GFP-tagged clathrin light chain (GFP-CLC), but this was significantly reduced in Shank knockdown neurons (44% ± 2%, $p < 0.001$; Figures 3B and 3C). The density of GFP-CLC puncta along the dendrite was not different between control and Shank knockdown neurons (Figure S3A). Also, immunolabeled clathrin puncta were less frequently associated with synapses labeled with anti-Homer1b/c in Shank knockdown neurons compared to control neurons (untransfected: 72% ± 3%, control: 78% ± 3%, mirShank: 39% ± 5%; Figure 3D). The synaptic distribution of Homer1c-mCherry was not altered in Shank knockdown neurons (Figures S3B–S3D).

To test whether specific interaction motifs in SHANK2 are required for coupling the EZ to the PSD, we determined the fraction of EZ-positive (EZ^+) PSDs in Shank knockdown neurons co-expressing miRNA-resistant wild-type (WT) SHANK2 (mirShank::SHANK2-WT; WT) and mutant forms of SHANK2 that lack the Dynamin2 (mirShank::SHANK2-ΔDYN; ΔDYN), Cortactin (mirShank::SHANK2-ΔCOR; ΔCOR), or Homer1b/c (mirShank::SHANK2-P1035L; P1035L) bindings sites. All of the mutants were effectively targeted to the PSD and did not alter synapse density (Figures S3E–S3G), and were used as a marker of the PSD. Whereas re-expression of SHANK2-WT completely restored the fraction of EZ^+ PSDs to control levels, the Dynamin2, Cortactin, or Homer1c binding site mutants were unable to rescue this (WT: 66% ± 3%, ΔDYN: 40.1% ± 3%, ΔCOR: 35.3% ± 3%, P1035L: 42.2% ± 2%, $p < 0.001$; Figures 3E and 3F). However, complete removal of the SHANK2 PDZ domain (ΔPDZ) did not alter the ability of SHANK2 to rescue the fraction of EZ^+ PSDs (66.1% ± 2%, $n = 10$; Figures 3E and 3F). Also, the overall density of GFP-CLC puncta in dendrites was not different between conditions (Figure S3H). Thus, these data indicate that SHANK2 binding to Homer1b/c, Cortactin, and Dynamin2 contribute to positioning the EZ close to the PSD. Similar to SHANK2, re-expression of Shank1 and SHANK3 completely restored the fraction of EZ^+ PSDs (Shank1: 70.3% ± 3%, SHANK2: 73.6% ± 2%, SHANK3: 72.9% ± 2%; Figures S3I and S3J).

Among the numerous *de novo* mutations in the *SHANK2* gene identified in individuals with ASD, one particular nonsense mutation in *SHANK2* (T1127M) is located in the core of the Dynamin2 binding site (Berkel et al., 2010). We confirmed that this SHANK2 variant was efficiently targeted to synapses (Figure S3E) (Berkel et al., 2012), but this single point mutation rendered SHANK2 unable to rescue the loss of EZ^+ PSDs (45.2% ± 4%, $p < 0.001$; Figures 3E and 3F). Another *de novo* mutation found in *SHANK2* in an individual with autism (L1008P1009dup; LPdup) was still able to rescue the loss of EZ-associated PSDs (66.9% ± 3%; Figures 3E and 3F).

To further test whether Shank proteins promote the local endocytosis of mGluR5, we determined the localization of internalized myc-mGluR5 with respect to the PSD. The fraction of synapses that overlapped with internalized mGluR5 puncta was significantly reduced in Shank knockdown neurons (control: 50.2% ± 4%, mirShank: 29.0% ± 3%, $p < 0.01$; Figures 3G and 3H). In neurons re-expressing SHANK2-WT, this was completely restored, while SHANK2 mutants deficient in binding Homer1b/c, Cortactin, or Dynamin2 were unable to rescue this (WT: 55.8% ± 4%, ΔDYN: 32.1% ± 2%, ΔCOR: 35.7% ± 5%, P1035L: 33.4% ± 4%, $p < 0.001$; Figures 3I and 3J). Also, in neurons re-expressing the ASD-associated SHANK2-T1127M mutant, there was a significant reduction in synapse-associated mGluR5 puncta (32% ± 4%, $p < 0.001$; Figures 3I and 3J). However, re-expression of SHANK2-ΔPDZ and the ASD-associated SHANK2-LPdup mutant did not alter the ability of SHANK2 to rescue this (ΔPDZ: 56.4% ± 4%, LPdup: 61.8% ± 4%; Figures 3I and 3J). Thus, Shank proteins spatially restrict the endocytosis of mGluR5 to perisynaptic sites by coupling the EZ to the PSD.

The EZ Mediates Local mGluR5 Recycling

The reduction in EZ-associated synapses and the decrease in mGluR5 internalization in Shank knockdown neurons suggest that mGluR5 internalizes through the spine EZ coupled to the PSD via Shank intermediates. To further test whether mGluR5 can undergo recycling and whether this is facilitated by the EZ, we performed an antibody-based recycling assay to specifically label the recycled pool of receptors (Lu et al., 2007). The levels of recycled mGluR5 were significantly higher at EZ^+ PSDs, with almost no detectable recycling at EZ^- PSDs (30 min: EZ^+: 1.2 ± 0.3, EZ^-: 0.8 ± 0.2; 60 min: EZ^+: 1.6 ± 0.2, EZ^-: 0.9 ± 0.1, $p < 0.05$; Figures 3K and 3L), consistent with the model that mGluR5 is internalized through the EZ to undergo local capture and recycling, reminiscent of AMPAR recycling (Lu et al., 2007).

Shank Proteins Control Local Trafficking of mGluR5 in Spines

We found that Shank knockdown specifically reduced the agonist-induced internalization of mGluR5 in spines, but not in dendrites, and predicted that disrupting the coupling between the EZ and the PSD would favor mGluR5 internalization at extrasynaptic sites. The density of internalized mGluR5 puncta at the dendritic shaft under basal conditions ($t = 0$ min) was similar in control and Shank knockdown neurons (Figure S4A) and showed a similar increase 5 min after the application of DHPG (Figures 4A and 4B). However, 10 min after treatment with DHPG, the density of internalized mGluR5 puncta at the dendritic shaft was significantly increased in Shank knockdown neurons compared to $t = 0$ min (0 min: 1 ± 0.09, 10 min: 2.0 ± 0.2, $p < 0.001$) and significantly different from control neurons (10 min: 1.2 ± 0.1, $p = 0.01$) (Figures 4A and 4B). This increase returned to control levels 20 min after treatment. Thus, in the absence of Shanks, activated receptors diffuse away from the synapse to internalize at extrasynaptic sites. This is expected to lead to a progressive

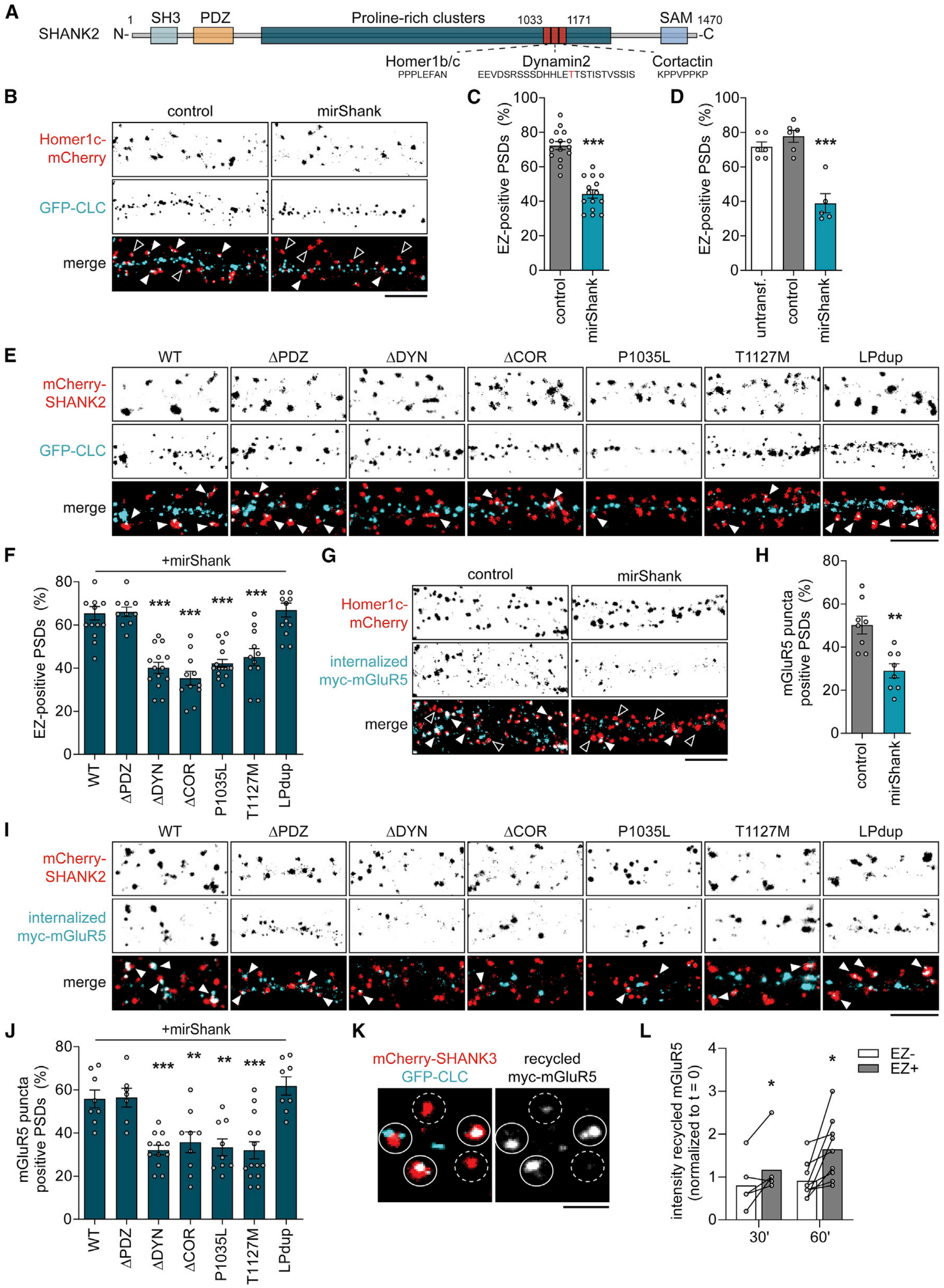

(legend on next page)

reduction in surface mGluR5 levels at the synapse. Enrichment of SEP-mGluR5 in spines was significantly reduced in Shank knockdown neurons, and fully rescued by re-expression of Shank1, SHANK2, and SHANK3 (control: 1.5 ± 0.1, mirShank: 1.1 ± 0.04, Shank1: 1.4 ± 0.04, SHANK2: 1.5 ± 0.1, SHANK3: 1.4 ± 0.04, $p < 0.001$; Figure S4B). Immunoblotting showed no reduction in mGluR5 proteins levels in Shank knockdown neurons (Figures S4C and S4D). However, the immunolabeling of mGluR5 showed that total levels of endogenous mGluR5 were reduced in Shank knockdown neurons (control: 1.0 ± 0.05, mirShank: 0.8 ± 0.03, $p < 0.01$; Figures S4E and S4F), which has been previously reported in Shank3 knockdown neurons (Verpelli et al., 2011). Thus, disrupted mGluR5 recycling in Shank knockdown neurons leads to a reduction in the density of mGluR5 at the synaptic membrane.

Shank proteins are large multi-domain scaffolding proteins and have been suggested to anchor mGluR5 at the synapse through interactions with Homer1b/c (Tu et al., 1999). The reduced surface levels of mGluR5 in spines and the total levels of endogenous mGluR5 in Shank knockdown neurons could also be explained by a reduction in receptor binding sites at the PSD, modulating the stability of mGluR5. However, using fluorescence recovery after photobleaching (FRAP) experiments, we found that the rate of recovery and the total recovery of Venus-mGluR5 were similar in control and Shank knockdown neurons (Figures 4C and 4D), suggesting that Shank proteins do not directly contribute to the anchoring of mGluR5 at synaptic sites.

mGluR5-Mediated Calcium and ERK1/2 Signaling Is Abrogated in Shank Knockdown Neurons

The density of mGluR5 on the membrane controls the activation of downstream signaling pathways (Choi et al., 2011; Nash et al., 2002) that can trigger the oscillatory release of Ca^{2+} from internal stores (Kawabata et al., 1996) and activate the extracellular signal-regulated kinase (ERK1/2) pathway (Mao et al., 2005). To test the functional impact of the defect in mGluR5 trafficking in Shank knockdown neurons, we measured DHPG-induced Ca^{2+} oscillations using the fluorescent Ca^{2+} reporter GCaMP6s (Chen et al., 2013). Consistent with previous reports, we found that DHPG triggered the immediate onset of robust Ca^{2+} oscillations (Figures 4E and 4F). DHPG-induced oscillations were completely blocked by the addition of the specific mGluR5 antagonist 2-methyl-6-(phenylethynyl)pyridine (MPEP) (DHPG: 17.3 ± 1.3 mHz and DHPG + MPEP: 3.5 ± 1.4 mHz, $p < 0.001$; Figures 4F and 4G), confirming that these oscillations are mediated by the activation of mGluR5. We found that the frequency of DHPG-induced calcium peaks was significantly reduced in Shank knockdown neurons (control 21.6 ± 3.1 mHz, mirShank 12.6 ± 1.5 mHz, $p < 0.01$; Figures 4H–4J). Furthermore, we compared DHPG-induced phosphorylation of ERK1/2 (pERK1/2) in control and Shank knockdown neurons. Incubation with DHPG for 10 and 30 min increased pERK1/2 shown by the immunolabeling of pERK1/2 in a population of control neurons, which was significantly reduced in Shank knockdown neurons after 30 min of DHPG treatment (10 min DHPG: control: 1.4 ± 0.15, mirShank: 0.95 ± 0.08; 30 min DHPG: control: 1.47 ± 0.16, mirShank: 0.89 ± 0.09, $p < 0.01$; Figures 4K and 4L). Under basal conditions (non-treated [NT]) the levels of pERK1/2 were similar between control and Shank knockdown neurons (NT: control: 1.00 ± 0.10, mirShank: 0.94 ± 0.11; Figures 4J and 4K). These results indicate that Shank regulates mGluR5 signaling, substantiating an involvement of aberrant receptor trafficking in animal models of ASD with implicated deregulation of mGluR5 signaling.

DISCUSSION

Modulation of glutamatergic signaling by group I mGluRs is essential for proper synaptic transmission and plasticity, and deregulated mGluR signaling is broadly held to underlie the molecular pathology of neurodevelopmental disorders (Lüscher and Huber, 2010). However, fundamental aspects of mGluR signaling and trafficking at excitatory synapses are still poorly

Figure 3. Shanks Couple the EZ to the PSD to Control mGluR5 Trafficking in Spines

(A) Domain structure of SHANK2. Proline-rich binding motifs for Homer1b/c, Dynamin2, and Cortactin are indicated.

(B) Representative images of dendrites co-expressing GFP-CLC (cyan) and Homer1c-mCherry (red) in control and mirShank neurons. Indicated are examples of EZ^+ (filled arrowhead) and EZ^- (open arrowheads) PSDs. Scale bar, 5 μm.

(C) Quantification of the percentage of PSDs associated with a GFP-CLC marked EZ in control (n = 15) and mirShank (n = 15) neurons.

(D) Quantification of PSDs associated with endogenous anti-clathrin in untransfected (n = 6) and transfected control (n = 6) and mirShank (n = 5) neurons.

(E) Representative images of dendrites co-expressing mCherry-tagged SHANK2 rescue constructs (red) and GFP-CLC (cyan). Scale bar, 5 μm.

(F) Quantification of the percentage of EZ^+ PSDs in neurons co-expressing mCherry-tagged SHANK2 rescue constructs (WT: n = 14, ΔPDZ: n = 10, ΔDYN: n = 14, ΔCOR: n = 11, P1035L: n = 15, T1127M: n = 11, LPdup: n = 13).

(G) Representative images showing internalized myc-mGluR5 (cyan) puncta 30 min after the application of DHPG in dendrites co-expressing Homer1c-mCherry (red) as a PSD marker, in control and mirShank neurons. Indicated are examples of internalized mGluR5 puncta-positive PSDs (filled arrowhead) and mGluR5 puncta-negative PSDs (open arrowheads). Scale bar, 5 μm.

(H) Quantification of the percentage of internalized mGluR5 puncta-positive PSDs in control (n = 8) and mirShank (n = 8) neurons.

(I) Representative images of dendrites co-expressing mCherry-tagged SHANK2 rescue constructs (red) and internalized myc-mGluR5 (cyan) 30 min after the application of DHPG. Scale bar, 5 μm.

(J) Quantification of the percentage of internalized mGluR5 puncta-positive PSDs in neurons co-expressing mCherry-tagged SHANK2 rescue constructs (WT: n = 8, ΔPDZ: n = 7, ΔDYN: n = 12, ΔCOR: n = 9, P1035L: n = 9, T1127M: n = 14, LPdup: n = 8).

(K) Representative image of recycled myc-mGluR5 (right panel) at EZ^+ PSDs (white circles) and at EZ^- PSDs (white dashed circles). EZs are marked by GFP-CLC (cyan) and PSDs are marked by mCherry-SHANK3 (red) (left panel). Scale bar, 2 μm.

(L) Quantification of the signal intensity of recycled myc-mGluR5 at EZ^- and EZ^+ PSDs after 30 (n = 6) and 60 (n = 9) min of recycling. Data are normalized to myc-mGluR5 intensity at t = 0 min.

Data are represented as means ± SEMs. *$p < 0.05$, **$p < 0.01$, ***$p < 0.001$.

OPEN ACCESS
CellPress

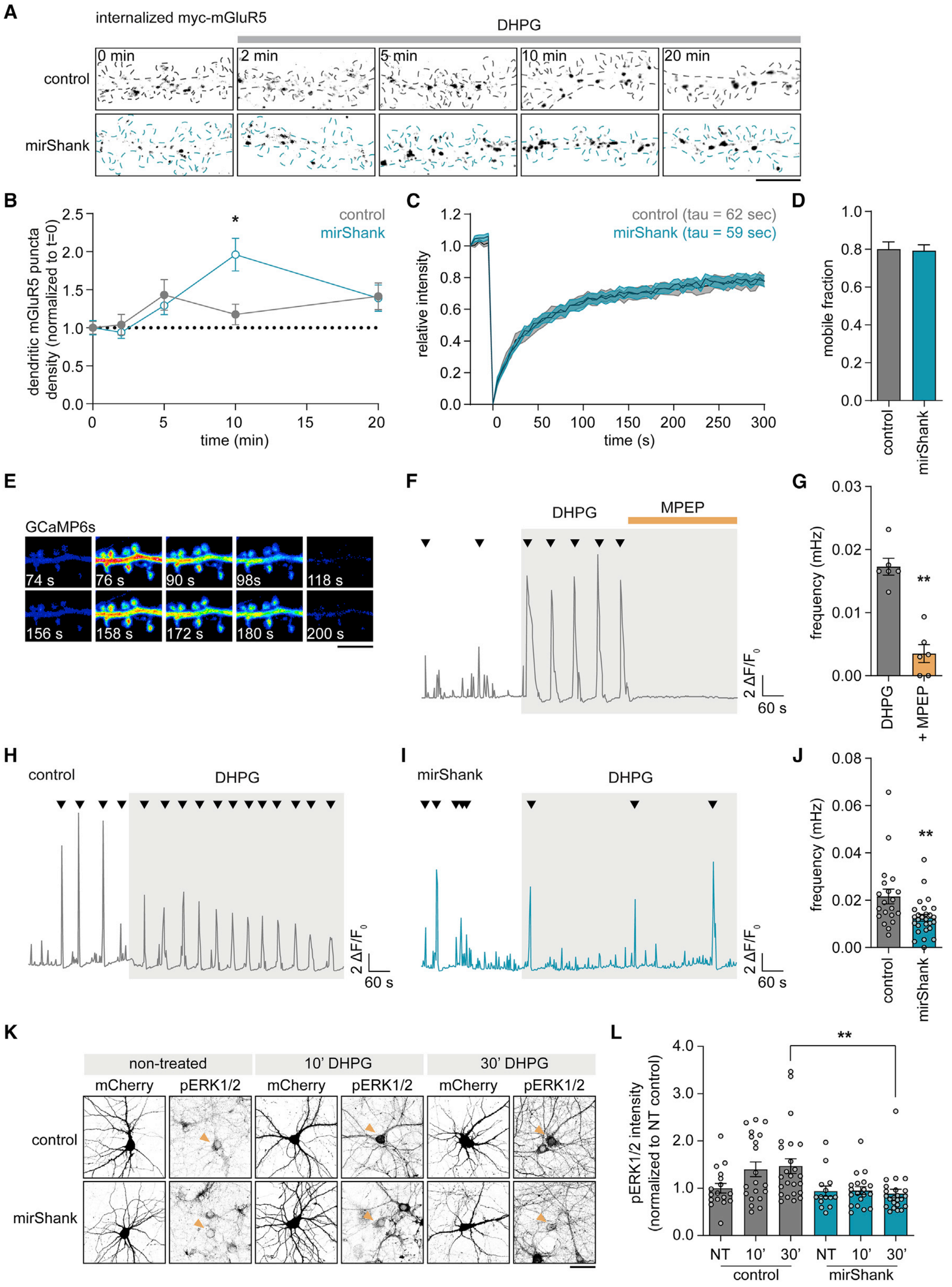

Figure 4. Shanks Control mGluR5-Mediated Calcium Signaling and ERK1/2 Activity

(A) Dendrite stained for internalized myc-mGluR5 at different time points after DHPG stimulation in control (gray outline; top panels) and mirShank (blue outline; bottom panels) neurons. Scale bar, 5 μm.

(legend continued on next page)

understood. Here, we present a model in which coupling of the EZ to the PSD by Shank proteins enables local recycling of mGluRs, allowing the synapse to balance the density of mGluRs at the membrane to efficiently modulate neuronal functioning.

Our data show that Shank proteins selectively regulate activity-induced internalization of mGluR5 in spines. While DHPG-induced mGluR5 internalization is greatly affected in Shank knockdown neurons, in the absence of stimulation, the levels of mGluR5 remain relatively constant in both control and Shank knockdown neurons. This suggests that in the absence of Shanks, constitutive internalization of mGluR5 is not affected and continues to replace surface receptors. Thus, in the absence of efficient PSD-EZ coupling, synaptic receptors now escape this local endocytic sink and become internalized and recycled at extrasynaptic sites, slowly depleting the synaptic pool of receptors. We found a significant increase in agonist-induced mGluR5 internalization in dendrites and a significant decrease in surface mGluR5 levels in the spines of Shank knockdown neurons. This also suggests that dendritic internalization of mGluR5 is regulated independent of Shanks. Moreover, blocking Dynamin activity did not alter dendritic mGluR5 internalization, indicating that the internalization of dendritic receptors is regulated by different mechanisms. We consistently found a small reduction in SEP-mGluR5 signal in the absence of agonists, both in spines and dendrites. This could reflect the constitutive internalization of mGluR5, but ongoing recycling and lateral diffusion of receptors make it hard to interpret this directly. Nevertheless, previous studies found that mGluR5 undergoes constitutive internalization at a similar rate, but that this process is independent of clathrin and Dynamin activity (Fourgeaud et al., 2003), and has been suggested to be mediated by the caveolin-mediated internalization pathway (Francesconi et al., 2009). Our results are consistent with the notion that the EZ captures synaptic receptors through spatially restricted, clathrin-mediated endocytosis and recycling, allowing the synapse to autonomously control its receptor content (Czöndör et al., 2012).

The reduction in surface mGluR5 levels in spines in Shank knockdown neurons was functionally reflected in a decrease in mGluR5-mediated calcium responses and ERK1/2 activation. Our experiments were focused on mGluR5, but we cannot exclude that other synaptic receptors or ion channels undergo aberrant trafficking when the EZ is uncoupled from the PSD. Glutamatergic transmission in general is reduced in Shank knockdown neurons (Arons et al., 2012; Duffney et al., 2013; Verpelli et al., 2011), Shank knockout mouse models (Bozdagi et al., 2010; Duffney et al., 2015; Kouser et al., 2013; Schmeisser et al., 2012), and neurons expressing ASD-associated Shank mutations (Lee et al., 2019). Thus, disrupting the link between the PSD and the EZ could have much broader effects on the composition of the synaptic membrane and glutamatergic transmission.

Our results indicate that all three Shank isoforms, which share a similar domain structure, recruit important components of the endocytic machinery to the PSD. The interaction between Shank and Homer1b/c confers a direct molecular link to the EZ through Dynamin3. Abrogating this interaction through directed mutation (our data) or through dominant-negative approaches (Lu et al., 2007) significantly impairs EZ positioning. Shank proteins also seem to recruit Dynamin2 to the EZ, which likely provides the GTPase activity necessary for vesicle scission. We found that the Cortactin-binding motif in SHANK2 was also required for efficient mGluR5 internalization. Cortactin can also bind Dynamin2 and -3 directly (Gray et al., 2003) and has been implicated in endocytosis and endolysomal sorting of AMPARs (Parkinson et al., 2018). The expression of the Shank triple knockdown construct leads to a strong reduction in total Shank levels, but leaves ~20% of total Shank levels intact (MacGillavry et al., 2016). Thus, we cannot exclude that remaining Shank proteins still recruit other interacting proteins that contribute to the trafficking of synaptic receptors.

Consistent with our results that Shank proteins control mGluR trafficking and function, recent studies show that deficits in social behavior caused by the loss of Shank function could be rescued by group I mGluR positive allosteric modulators (Bariselli et al., 2016; Vicidomini et al., 2017). However, even though deregulated receptor functioning at excitatory synapses has been implicated to underlie physiological deficits in many disease models, the molecular mechanisms underlying this have not been resolved. Our results indicate that Shank proteins do not directly anchor receptors at the synapse but provide a stable molecular framework that permits the local uptake and trafficking of receptors via the EZ, thereby governing a stable pool of synaptic receptors. That the ASD-associated T1127M mutation in SHANK2 disrupts this process further underlines the relevance of understanding the functional relation between Shank

(B) Quantification of internalized myc-mGluR5 puncta density in the dendritic shaft at different time points after DHPG stimulation in control (n = 18–24) and mirShank (n = 18–27) neurons, normalized to t = 0 per condition.
(C) FRAP analysis of Venus-mGluR5 in spines of control (gray; n = 38 spines) and mirShank (blue; n = 38 spines) neurons.
(D) Quantification of the mobile fraction of Venus-mGluR5 in spines of control and mirShank neurons.
(E) Example of a dendrite expressing GCaMP6s stimulated with DHPG. Scale bar, 5 μm.
(F) Oscillatory response of GCaMP6s signal in response to the application of DHPG (gray; n = 22) and DHPG + MPEP (orange; n = 26).
(G) Quantification of the frequency (millihertz) of GCaMP6s oscillations in response to DHPG and DHPG + MPEP (n = 6).
(H and I) Oscillatory response of GCaMP6s signal in response to DHPG in control (gray) (H) and mirShank (blue) (I) neurons.
(J) Quantification of the frequency (millihertz) of GCaMP6s oscillations in response to DHPG in control (n = 20) and mirShank (n = 27) neurons.
(K) Examples of control (top panels) and mirShank (bottom panels) neurons immunolabeled for anti-pERK1/2 in non-treated (NT) and treated neurons with DHPG for 10 min (10') or 30 min (30'). Orange arrowheads indicate the mCherry expressing control and mirShank neurons used for quantification. Scale bar, 50 μm.
(L) Quantification of the average anti-pERK1/2 fluorescence intensity in the cell bodies of the transfected neurons of control (n = 17–24) and mirShank (n = 14–23) neurons with indicated treatment. Data are normalized to the average intensity of the NT control condition.
Data are represented as means ± SEMs. *p < 0.05, **p < 0.01, and ***p < 0.001.

proteins and mGluR signaling in the context of human neurodevelopmental disorders.

STAR★METHODS

Detailed methods are provided in the online version of this paper and include the following:

- KEY RESOURCES TABLE
- LEAD CONTACT AND MATERIALS AVAILABILITY
- EXPERIMENTAL MODEL AND SUBJECT DETAILS
  - Animals
  - Primary Neuronal Cultures and Transfections
- METHOD DETAILS
  - DNA Constructs
  - Confocal Imaging
  - Antibody Feeding Assay
  - Endocytic Zone Associated PSDs
  - Receptor Recycling Assay
  - Endogenous mGluR5 Protein Levels
  - ERK1/2 Phosphorylation Assay
  - Live-Cell Imaging
  - Live-Cell Imaging of SEP-Tagged mGluR5
  - SEP pH Sensitivity Assay
  - Live-Cell Imaging of AcidiFluor ORANGE Halo-Tagged mGluR5
  - Fluorescence Recovery after Photobleaching
  - Calcium Imaging
  - Western Blot and Imaging
- QUANTIFICATION AND STATISTICAL ANALYSIS
  - Quantification of Endocytic Zone Associated PSDs
  - Quantification of Internalized mGluR5 Puncta in Spines and Dendrites
  - Quantification of SEP-mGluR5 Internalization in Spines and Dendrites
  - Quantification of AcidiFluor ORANGE Halo-Tagged mGluR5 Acidification in Spines and Dendrites
  - Quantification of Spine Enrichment
  - Quantification of Immunofluorescence of Endogenous mGluR5
  - Quantification of FRAP Experiment
  - Quantification of Calcium Experiment
  - Quantification of ERK1/2 Phosphorylation Assay
  - Statistical Analysis
- DATA AND CODE AVAILABILITY

SUPPLEMENTAL INFORMATION

Supplemental Information can be found online at https://doi.org/10.1016/j.celrep.2019.08.102.

ACKNOWLEDGMENTS

We would like to thank the MacGillavry lab for helpful discussions. This work was supported by the Netherlands Organization for Scientific Research (NWO-ALW-VENI to H.D.M. and the Graduate Program of Quantitative Biology and Computational Life Sciences to N.S.), the Federation of European Biochemical Societies (FEBS Return-to-Europe Fellowship), the European Research Council (ERC-StG 716011), and the Brain and Behavior Research Foundation (NARSAD Young Investigator Award) to H.D.M.

AUTHOR CONTRIBUTIONS

Conceptualization, Methodology, Validation, & Formal Analysis, N.S. and H.D.M.; Investigation, N.S., L.A.E.C., M.L.W., and H.D.M.; Resources, H.D.M., C.C.H., and T.A.B.; Writing – Original Draft & Editing, N.S. and H.D.M.; Writing – Review, C.C.H. and T.A.B.; Visualization, N.S. and H.D.M.; Supervision, H.D.M.; Funding Acquisition, H.D.M.

DECLARATION OF INTERESTS

The authors declare no competing interests.

Received: February 8, 2019
Revised: July 25, 2019
Accepted: August 29, 2019
Published: October 8, 2019

REFERENCES

Arons, M.H., Thynne, C.J., Grabrucker, A.M., Li, D., Schoen, M., Cheyne, J.E., Boeckers, T.M., Montgomery, J.M., and Garner, C.C. (2012). Autism-associated mutations in ProSAP2/Shank3 impair synaptic transmission and neurexin-neuroligin-mediated transsynaptic signaling. J. Neurosci. *32*, 14966–14978.

Bariselli, S., Tzanoulinou, S., Glangetas, C., Prévost-Solié, C., Pucci, L., Viguié, J., Bezzi, P., O'Connor, E.C., Georges, F., Lüscher, C., and Bellone, C. (2016). SHANK3 controls maturation of social reward circuits in the VTA. Nat. Neurosci. *19*, 926–934.

Berkel, S., Marshall, C.R., Weiss, B., Howe, J., Roeth, R., Moog, U., Endris, V., Roberts, W., Szatmari, P., Pinto, D., et al. (2010). Mutations in the SHANK2 synaptic scaffolding gene in autism spectrum disorder and mental retardation. Nat. Genet. *42*, 489–491.

Berkel, S., Tang, W., Treviño, M., Vogt, M., Obenhaus, H.A., Gass, P., Scherer, S.W., Sprengel, R., Schratt, G., and Rappold, G.A. (2012). Inherited and de novo SHANK2 variants associated with autism spectrum disorder impair neuronal morphogenesis and physiology. Hum. Mol. Genet. *21*, 344–357.

Blanpied, T.A., Scott, D.B., and Ehlers, M.D. (2002). Dynamics and regulation of clathrin coats at specialized endocytic zones of dendrites and spines. Neuron *36*, 435–449.

Bozdagi, O., Sakurai, T., Papapetrou, D., Wang, X., Dickstein, D.L., Takahashi, N., Kajiwara, Y., Yang, M., Katz, A.M., Scattoni, M.L., et al. (2010). Haploinsufficiency of the autism-associated Shank3 gene leads to deficits in synaptic function, social interaction, and social communication. Mol. Autism *1*, 15.

Chen, T.W., Wardill, T.J., Sun, Y., Pulver, S.R., Renninger, S.L., Baohan, A., Schreiter, E.R., Kerr, R.A., Orger, M.B., Jayaraman, V., et al. (2013). Ultrasensitive fluorescent proteins for imaging neuronal activity. Nature *499*, 295–300.

Choi, K.Y., Chung, S., and Roche, K.W. (2011). Differential binding of calmodulin to group I metabotropic glutamate receptors regulates receptor trafficking and signaling. J. Neurosci. *31*, 5921–5930.

Cochoy, D.M., Kolevzon, A., Kajiwara, Y., Schoen, M., Pascual-Lucas, M., Lurie, S., Buxbaum, J.D., Boeckers, T.M., and Schmeisser, M.J. (2015). Phenotypic and functional analysis of SHANK3 stop mutations identified in individuals with ASD and/or ID. Mol. Autism *6*, 23.

Cunha-Ferreira, I., Chazeau, A., Buijs, R.R., Stucchi, R., Will, L., Pan, X., Adolfs, Y., van der Meer, C., Wolthuis, J.C., Kahn, O.I., et al. (2018). The HAUS Complex Is a Key Regulator of Non-centrosomal Microtubule Organization during Neuronal Development. Cell Rep. *24*, 791–800.

Czöndör, K., Mondin, M., Garcia, M., Heine, M., Frischknecht, R., Choquet, D., Sibarita, J.B., and Thoumine, O.R. (2012). Unified quantitative model of AMPA receptor trafficking at synapses. Proc. Natl. Acad. Sci. USA *109*, 3522–3527.

Dhami, G.K., and Ferguson, S.S. (2006). Regulation of metabotropic glutamate receptor signaling, desensitization and endocytosis. Pharmacol. Ther. *111*, 260–271.

Duffney, L.J., Wei, J., Cheng, J., Liu, W., Smith, K.R., Kittler, J.T., and Yan, Z. (2013). Shank3 deficiency induces NMDA receptor hypofunction via an actin-dependent mechanism. J. Neurosci. *33*, 15767–15778.

Duffney, L.J., Zhong, P., Wei, J., Matas, E., Cheng, J., Qin, L., Ma, K., Dietz, D.M., Kajiwara, Y., Buxbaum, J.D., and Yan, Z. (2015). Autism-like Deficits in Shank3-Deficient Mice Are Rescued by Targeting Actin Regulators. Cell Rep. *11*, 1400–1413.

Esteves da Silva, M., Adrian, M., Schätzle, P., Lipka, J., Watanabe, T., Cho, S., Futai, K., Wierenga, C.J., Kapitein, L.C., and Hoogenraad, C.C. (2015). Positioning of AMPA Receptor-Containing Endosomes Regulates Synapse Architecture. Cell Rep. *13*, 933–943.

Falcón-Pérez, J.M., Nazarian, R., Sabatti, C., and Dell'Angelica, E.C. (2005). Distribution and dynamics of Lamp1-containing endocytic organelles in fibroblasts deficient in BLOC-3. J. Cell Sci. *118*, 5243–5255.

Fourgeaud, L., Bessis, A.S., Rossignol, F., Pin, J.P., Olivo-Marin, J.C., and Hémar, A. (2003). The metabotropic glutamate receptor mGluR5 is endocytosed by a clathrin-independent pathway. J. Biol. Chem. *278*, 12222–12230.

Francesconi, A., Kumari, R., and Zukin, R.S. (2009). Regulation of group I metabotropic glutamate receptor trafficking and signaling by the caveolar/lipid raft pathway. J. Neurosci. *29*, 3590–3602.

Gray, N.W., Fourgeaud, L., Huang, B., Chen, J., Cao, H., Oswald, B.J., Hémar, A., and McNiven, M.A. (2003). Dynamin 3 is a component of the postsynapse, where it interacts with mGluR5 and Homer. Curr. Biol. *13*, 510–515.

Gross, G.G., Junge, J.A., Mora, R.J., Kwon, H.B., Olson, C.A., Takahashi, T.T., Liman, E.R., Ellis-Davies, G.C., McGee, A.W., Sabatini, B.L., et al. (2013). Recombinant probes for visualizing endogenous synaptic proteins in living neurons. Neuron *78*, 971–985.

Hoogenraad, C.C., Popa, I., Futai, K., Martinez-Sanchez, E., Wulf, P.S., van Vlijmen, T., Dortland, B.R., Oorschot, V., Govers, R., Monti, M., et al. (2010). Neuron specific Rab4 effector GRASP-1 coordinates membrane specialization and maturation of recycling endosomes. PLoS Biol. *8*, e1000283.

Isa, M., Asanuma, D., Namiki, S., Kumagai, K., Kojima, H., Okabe, T., Nagano, T., and Hirose, K. (2014). High-throughput screening system to identify small molecules that induce internalization and degradation of HER2. ACS Chem. Biol. *9*, 2237–2241.

Kawabata, S., Tsutsumi, R., Kohara, A., Yamaguchi, T., Nakanishi, S., and Okada, M. (1996). Control of calcium oscillations by phosphorylation of metabotropic glutamate receptors. Nature *383*, 89–92.

Kessels, M.M., Engqvist-Goldstein, A.E., Drubin, D.G., and Qualmann, B. (2001). Mammalian Abp1, a signal-responsive F-actin-binding protein, links the actin cytoskeleton to endocytosis via the GTPase dynamin. J. Cell Biol. *153*, 351–366.

Kouser, M., Speed, H.E., Dewey, C.M., Reimers, J.M., Widman, A.J., Gupta, N., Liu, S., Jaramillo, T.C., Bangash, M., Xiao, B., et al. (2013). Loss of predominant Shank3 isoforms results in hippocampus-dependent impairments in behavior and synaptic transmission. J. Neurosci. *33*, 18448–18468.

Lee, J.H., Lee, J., Choi, K.Y., Hepp, R., Lee, J.Y., Lim, M.K., Chatani-Hinze, M., Roche, P.A., Kim, D.G., Ahn, Y.S., et al. (2008). Calmodulin dynamically regulates the trafficking of the metabotropic glutamate receptor mGluR5. Proc. Natl. Acad. Sci. USA *105*, 12575–12580.

Lee, K., Vyas, Y., Garner, C.C., and Montgomery, J.M. (2019). Autism-associated Shank3 mutations alter mGluR expression and mGluR-dependent but not NMDA receptor-dependent long-term depression. Synapse *73*, e22097.

Lois, C., Hong, E.J., Pease, S., Brown, E.J., and Baltimore, D. (2002). Germline transmission and tissue-specific expression of transgenes delivered by lentiviral vectors. Science *295*, 868–872.

Lu, J., Helton, T.D., Blanpied, T.A., Rácz, B., Newpher, T.M., Weinberg, R.J., and Ehlers, M.D. (2007). Postsynaptic positioning of endocytic zones and AMPA receptor cycling by physical coupling of dynamin-3 to Homer. Neuron *55*, 874–889.

Lüscher, C., and Huber, K.M. (2010). Group 1 mGluR-dependent synaptic long-term depression: mechanisms and implications for circuitry and disease. Neuron *65*, 445–459.

MacGillavry, H.D., Song, Y., Raghavachari, S., and Blanpied, T.A. (2013). Nanoscale scaffolding domains within the postsynaptic density concentrate synaptic AMPA receptors. Neuron *78*, 615–622.

MacGillavry, H.D., Kerr, J.M., Kassner, J., Frost, N.A., and Blanpied, T.A. (2016). Shank-cortactin interactions control actin dynamics to maintain flexibility of neuronal spines and synapses. Eur. J. Neurosci. *43*, 179–193.

Macia, E., Ehrlich, M., Massol, R., Boucrot, E., Brunner, C., and Kirchhausen, T. (2006). Dynasore, a cell-permeable inhibitor of dynamin. Dev. Cell *10*, 839–850.

Mao, L., Yang, L., Tang, Q., Samdani, S., Zhang, G., and Wang, J.Q. (2005). The scaffold protein Homer1b/c links metabotropic glutamate receptor 5 to extracellular signal-regulated protein kinase cascades in neurons. J. Neurosci. *25*, 2741–2752.

McNiven, M.A., Kim, L., Krueger, E.W., Orth, J.D., Cao, H., and Wong, T.W. (2000). Regulated interactions between dynamin and the actin-binding protein cortactin modulate cell shape. J. Cell Biol. *151*, 187–198.

Naisbitt, S., Kim, E., Tu, J.C., Xiao, B., Sala, C., Valtschanoff, J., Weinberg, R.J., Worley, P.F., and Sheng, M. (1999). Shank, a novel family of postsynaptic density proteins that binds to the NMDA receptor/PSD-95/GKAP complex and cortactin. Neuron *23*, 569–582.

Nash, M.S., Schell, M.J., Atkinson, P.J., Johnston, N.R., Nahorski, S.R., and Challiss, R.A. (2002). Determinants of metabotropic glutamate receptor-5-mediated Ca2+ and inositol 1,4,5-trisphosphate oscillation frequency. Receptor density versus agonist concentration. J. Biol. Chem. *277*, 35947–35960.

Ochoa, G.C., Slepnev, V.I., Neff, L., Ringstad, N., Takei, K., Daniell, L., Kim, W., Cao, H., McNiven, M., Baron, R., and De Camilli, P. (2000). A functional link between dynamin and the actin cytoskeleton at podosomes. J. Cell Biol. *150*, 377–389.

Okamoto, P.M., Gamby, C., Wells, D., Fallon, J., and Vallee, R.B. (2001). Dynamin isoform-specific interaction with the shank/ProSAP scaffolding proteins of the postsynaptic density and actin cytoskeleton. J. Biol. Chem. *276*, 48458–48465.

Parkinson, G.T., Chamberlain, S.E.L., Jaafari, N., Turvey, M., Mellor, J.R., and Hanley, J.G. (2018). Cortactin regulates endo-lysosomal sorting of AMPARs via direct interaction with GluA2 subunit. Sci. Rep. *8*, 4155.

Petrini, E.M., Lu, J., Cognet, L., Lounis, B., Ehlers, M.D., and Choquet, D. (2009). Endocytic trafficking and recycling maintain a pool of mobile surface AMPA receptors required for synaptic potentiation. Neuron *63*, 92–105.

Qualmann, B., Boeckers, T.M., Jeromin, M., Gundelfinger, E.D., and Kessels, M.M. (2004). Linkage of the actin cytoskeleton to the postsynaptic density via direct interactions of Abp1 with the ProSAP/Shank family. J. Neurosci. *24*, 2481–2495.

Rácz, B., Blanpied, T.A., Ehlers, M.D., and Weinberg, R.J. (2004). Lateral organization of endocytic machinery in dendritic spines. Nat. Neurosci. *7*, 917–918.

Rosendale, M., Jullié, D., Choquet, D., and Perrais, D. (2017). Spatial and Temporal Regulation of Receptor Endocytosis in Neuronal Dendrites Revealed by Imaging of Single Vesicle Formation. Cell Rep. *18*, 1840–1847.

Scheefhals, N., and MacGillavry, H.D. (2018). Functional organization of postsynaptic glutamate receptors. Mol. Cell. Neurosci. *91*, 82–94.

Schmeisser, M.J., Ey, E., Wegener, S., Bockmann, J., Stempel, A.V., Kuebler, A., Janssen, A.L., Udvardi, P.T., Shiban, E., Spilker, C., et al. (2012). Autistic-like behaviours and hyperactivity in mice lacking ProSAP1/Shank2. Nature *486*, 256–260.

Tu, J.C., Xiao, B., Naisbitt, S., Yuan, J.P., Petralia, R.S., Brakeman, P., Doan, A., Aakalu, V.K., Lanahan, A.A., Sheng, M., and Worley, P.F. (1999). Coupling of mGluR/Homer and PSD-95 complexes by the Shank family of postsynaptic density proteins. Neuron *23*, 583–592.

Verpelli, C., Dvoretskova, E., Vicidomini, C., Rossi, F., Chiappalone, M., Schoen, M., Di Stefano, B., Mantegazza, R., Broccoli, V., Böckers, T.M., et al. (2011). Importance of Shank3 protein in regulating metabotropic glutamate receptor 5 (mGluR5) expression and signaling at synapses. J. Biol. Chem. *286*, 34839–34850.

Vicidomini, C., Ponzoni, L., Lim, D., Schmeisser, M.J., Reim, D., Morello, N., Orellana, D., Tozzi, A., Durante, V., Scalmani, P., et al. (2017). Pharmacological enhancement of mGlu5 receptors rescues behavioral deficits in SHANK3 knock-out mice. Mol. Psychiatry *22*, 689–702.

STAR★METHODS

KEY RESOURCES TABLE

REAGENT or RESOURCE	SOURCE	IDENTIFIER
Antibodies		
Mouse anti-c-Myc (9E10) Monoclonal Antibody	Santa Cruz Biotechnology	Cat# sc-40; RRID: AB_627268
Human anti-EEA1 Antibody (clone 4114)	M. Fritzler	N/A
Rabbit anti-mGluR5 Antibody	Millipore	Cat# 06-451; RRID: AB_2313604
Rabbit anti-phospho-ERK1/2 Antibody	Cell Signaling	Cat# 9101; RRID: AB_331646
Mouse anti-PSD-95 Antibody	Neuromab	Cat# 75-028; RRID: AB_2292909
Mouse anti-Clathrin Heavy Chain (X22) Monoclonal Antibody	Thermo Fisher Scientific	Cat# MA1-065; RRID: AB_2083179
Rabbit anti-Homer1 Antibody	Synaptic Systems	Cat# 160 006; RRID: AB_2631222
Mouse anti-alpha-tubulin	Sigma-Aldrich	Cat# T6074; RRID: AB_477582
Goat anti-Human IgG (H+L) Secondary Antibody, Alexa Fluor 568	Thermo Fisher Scientific	Cat# A-21090; RRID: AB_2535746
Goat anti-Mouse IgG (H+L) Secondary Antibody, Alexa Fluor 647	Thermo Fisher Scientific	Cat# A-21236; RRID: AB_2535805
Goat anti-Mouse IgG (H+L) Secondary Antibody, Alexa Fluor 488	Thermo Fisher Scientific	Cat# A-11029; RRID: AB_2534088
Goat anti-Rabbit IgG (H+L) Secondary Antibody, Alexa Fluor 488	Thermo Fisher Scientific	Cat# A-11034; RRID: AB_2576217
Swine anti-mouse HRP-conjugated	Agilent	Cat# P0260; RRID: AB_263692
Goat Anti-Rabbit IgG Secondary Antibody, IRDye 680LT	LI-COR Biosciences	Cat# 827-11081; RRID: AB_10795015
Goat Anti-Mouse IgG Secondary Antibody, IRDye 800CW	LI-COR Biosciences	Cat# 827-08364; RRID: AB_10793856
Bacterial and Virus Strains		
Escherichia coli: BL21DE3	N/A	N/A
Chemicals, Peptides, and Recombinant Proteins		
Lipofectamine 2000	Thermo Fisher Scientific	Cat# 11668019
(S)-3,5-DHPG	Tocris	Cat# 805
Dynasore	Tocris	Cat# 2897
MPEP hydrochloride	Tocris	Cat# 1212
Tetrodotoxin citrate	Tocris	Cat# 1069
Polyvinyl alcohol mounting medium with DABCO®, antifading (Mowiol)	Sigma Aldrich	Cat# 10981
HaloTag® AcidiFluorORANGE Ligand	GORYO Chemical	Cat# GC310-01
Experimental Models: Cell Lines		
Human embryonic kidney 239T (HEK293T)	ATCC	Cat# CRL-3216; RRID: CVCL_0063
Experimental Models: Organisms/Strains		
Rattus norvegicus (Wistar; HanRj:WI)	RGD, Janvier labs	Cat# 13792727; RRID: RGD_13792727
Oligonucleotides		
See Table S1 for miRNA targeting sequences of Shank1, 2 and 3	N/A	N/A
ΔPDZ: deleted Thr254 – Thr348 with forward primer: ATTATTGAGGAGAAGAGGAATCTGGAC CCCG	This paper	N/A
ΔPDZ: deleted Thr254 – Thr348 with reverse primer: CTTCTCCTCAATAATGCAGTCA	This paper	N/A

(*Continued on next page*)

Continued

REAGENT or RESOURCE	SOURCE	IDENTIFIER
ΔDYN: deleted Glu1114 – Ser113 with forward primer: TTTGACGCCGTCGCCGACTCTGGGATC GAGACCCTGTCTTCCGAAGGTG	This paper	N/A
ΔDYN: deleted Glu1114 – Ser113 with reverse primer: CACATTCTCTCCACCTTCGGAAGACAG GGTCTCGATCCCAGAGTCGG	This paper	N/A
T1127M: mutagenesis with forward primer: AGCG ACCACCACCTCGAGATGACCAGCACTATCTCC ACCG	This paper	N/A
T1127M: mutagenesis with reverse primer: CGGT GGAGATAGTGCTGGTCATCTCGAGGTGGTGGT CGCT	This paper	N/A
L1008P1009: duplication with forward primer: GTGATTTTGCCATTGCCATTCCGCATCCCTCC	This paper	N/A
L1008P1009: duplication with reverse primer: GGG ATGCGGAATGGCAATGGCAAAATCACCGC	This paper	N/A
Recombinant DNA		
pRK5-Venus-mGluR5a	Dr. J. Perroy	N/A
pRK5-SEP-mGluR5a	This paper	N/A
pRK5-myc-mGluR5a	This paper	N/A
pRK5-Halo-mGluR5a	This paper	N/A
pRK5-SEP-mGluR1	This paper	clone image ID # 40080840
pSM155-Cer3	MacGillavry et al., 2016	N/A
pSM155-mCherry	This paper	N/A
pSM155-GFP	MacGillavry et al., 2016	N/A
pSM155-mirShank::Cer3	MacGillavry et al., 2016	N/A
pSM155-mirShank::mCherry	This paper	N/A
pSM155-mirShank::GFP	MacGillavry et al., 2016	N/A
pSM155-mirShank::mCherry-Shank1	This paper	N/A
pSM155-mirShank::mCherry-SHANK2	This paper and MacGillavry et al., 2016	N/A
pSM155-mirShank::mCherry-SHANK3	This paper	N/A
pSM155-mirShank::mCherry-SHANK2-ΔPDZ	This paper	N/A
pSM155-mirShank::mCherry-SHANK2-ΔDYN	This paper	N/A
pSM155-mirShank::mCherry-SHANK2-ΔCOR	This paper and MacGillavry et al., 2016	N/A
pSM155-mirShank::mCherry-SHANK2-P1035L	This paper and MacGillavry et al., 2016	N/A
pSM155-mirShank::mCherry-SHANK2-T1127M	This paper	N/A
pSM155-mirShank::mCherry-SHANK2-Lpdup	This paper	N/A
pcDNA3.1- mCherry-Shank2	Dr. Simone Berkel (Berkel et al., 2012)	N/A
mCherry-Shank3	Dr. M. Schmeisser (Cochoy et al., 2015)	N/A
pEGFP-C2-GFP-Clathrin-light-Chain	Dr. Mike Ehlers	
pmCherry-N1-Homer1c-mCherry	MacGillavry et al., 2013	N/A
GFP-Rab5	Hoogenraad et al., 2010	N/A
GFP-Rab11	Esteves da Silva et al., 2015	N/A
TfR-SEP	Hoogenraad et al., 2010	N/A
GFP-Rab7	Hoogenraad et al., 2010	N/A
pEGFP-N3-LAMP1-mGFP	Dr. Esteban Dell'Angelica (Falcón-Pérez et al., 2005)	http://addgene.org/34831; RRID: Addgene_34831
pEGFP-N1-Dynamin2-GFP	Dr. Pietro De Camilli (Ochoa et al., 2000)	N/A
pEGFP-N1-Dynamin2-K44A-GFP	Dr. Pietro De Camilli (Ochoa et al., 2000)	http://addgene.org/22301; RRID: Addgene_22301

(*Continued on next page*)

Continued

REAGENT or RESOURCE	SOURCE	IDENTIFIER
pGP-CMV-GcaMP6s	Dr. Douglas Kim (Chen et al., 2013)	http://addgene.org/40753; RRID: Addgene_40753
pCAG_PSD95.FingR-eGFP-CCR5TC	Dr. Don Arnold (Gross et al., 2013)	http://addgene.org/46295; RRID: Addgene_46295
FUGW	Dr. David Baltimore	http://addgene.og/14883 ; RRID: Addgene_14883
FUGW-mirShank-GFP	This paper	N/A
p.MDG2	Didier Trono	http://addgene.org/12259; RRID: Addgene_12259
psPAX2	Didier Trono	http://addgene.org/12260; RRID: Addgene_12260
Software and Algorithms		
ImageJ	NIH	https://imagej.nih.gov/ij/; RRID: SCR_003070
Fiji	Fiji	http://fiji.sc; RRID: SCR_002285
GraphPad Prism 8	GraphPad	https://www.graphpad.com/scientific-software/prism/; RRID: SCR_002798
Adobe Illustrator CC 2017	Adobe	https://www.adobe.com/products/illustrator.html; RRID: SCR_010279
MATLAB 2018a	MATLAB	https://www.mathworks.com/products/matlab/; RRID: SCR_001622

LEAD CONTACT AND MATERIALS AVAILABILITY

Plasmids generated in this study are available on request. Further information and requests for resources and reagents should be directed to and will be fulfilled by the Lead Contact, Harold MacGillavry (h.d.macgillavry@uu.nl).

EXPERIMENTAL MODEL AND SUBJECT DETAILS

Animals

All animal experiments were performed in compliance with the guidelines for the welfare of experimental animals issued by the Government of the Netherlands (Wet op de Dierproeven, 1996) and European regulations (Guideline 86/609/EEC). All animal experiments were approved by the Dutch Animal Experiments Review Committee (Dier Experimenten Commissie; DEC), performed in line with the institutional guidelines of Utrecht University.

Primary Neuronal Cultures and Transfections

Hippocampal cultures were prepared from embryonic day 18 (E18) Janvier Wistar rat brains (both genders) as described in Cunha-Ferreira et al. (2018). Dissociated neurons were plated on coverslips coated with poly-L-lysine (37.5 μg/ml, Sigma-Aldrich) and laminin (1.25 μg/ml, Roche Diagnostics) at a density of 100,000 neurons per well of a 12-well plate. Cultures were grown in Neurobasal medium (NB) supplemented with 2% B27 (GIBCO), 0.5 mM glutamine (GIBCO), 15.6 μM glutamate (Sigma-Aldrich), and 1% penicillin/ streptomycin at 37°C in 5% CO_2. At DIV14-18 neurons were transfected with indicated constructs using Lipofectamine 2000 (Invitrogen). Before transfection 260 μl conditioned medium was transferred to a new culture plate and replaced with 260 μl NB with 0.5 mM glutamine. For each well, 1.8 μg DNA was mixed with 3.3 μl Lipofectamine 2000 in 200 μl NB, incubated for 30 min at RT and added to the neurons. After 45 – 60 minutes, neurons were briefly washed with NB and transferred to the new culture plate with conditioned medium supplemented with 260 μl NB with B27, glutamine, penicillin/ streptomycin and kept at 37°C in 5% CO_2 for 2-4 days (for overexpression) or 5-7 days (for Shank knockdown).

METHOD DETAILS

DNA Constructs

The pRK5-SEP-mGluR5a, pRK5-Halo-mGluR5a and pRK5-myc-mGluR5a constructs were made using the pRK5-Venus-mGluR5a construct (a gift from Dr. Julie Perroy) as a template and the pRK5-SEP-mGluR1 construct was made by replacing mGluR5a with mGluR1 (clone image ID # 40080840). The human mCherry-SHANK2 expression plasmid was kindly provided by Berkel et al.

(2012). The pSM155-GFP (or Cerulean3; Cer3), Shank triple-knockdown construct pSM155-mirShank-GFP (or Cer3), and mirShank::GFP-SHANK2 wild-type (:: to indicate that the Shank miRNAs and GFP-tagged human SHANK2 are expressed simultaneously from a single expression cassette), mirShank::GFP-SHANK2-ΔCOR, and mirShank::GFP-SHANK2-P1035L mutant rescue constructs are described in MacGillavry et al. (2016). In these constructs GFP was replaced by mCherry (from pmCherry-N1, Invitrogen). To make the mirShank::mCherry-SHANK2-ΔDYN (lacking the 25-amino acid dynamin-binding domain; Glu1114 - Ser1138) (Okamoto et al., 2001), mirShank::mCherry-SHANK2-ΔPDZ (lacking the 95-amino acid PDZ domain, Thr254 - Thr348), mirShank::mCherry-SHANK2-L1008P1009dup and mirShank::mCherry-SHANK2-T1127M constructs, primers were designed containing the desired mutations and 10 – 15 bp overhangs for Gibson assembly (NEBuilder HiFi DNA assembly cloning kit). The rat Shank1 and human SHANK3 expression plasmids were a gift from Dr. Morgan Sheng and Dr. Michael Schmeisser (Cochoy et al., 2015), respectively, and used as a template to make the pSM155-mirShank::mCherry-Shank1 and pSM155-mirShank::mCherry-SHANK3 rescue constructs. Dynamin2-GFP and Dynamin2-K44A-GFP (Addgene plasmid # 22301) were a gift from Dr. Pietro De Camilli (Ochoa et al., 2000), and in both constructs GFP was replaced by mCherry. GFP-CLC (rat clathrin light chain A1) was a gift from Dr. Mike Ehlers, LAMP1-GFP was a gift from Dr. Esteban Dell'Angelica (Addgene plasmid # 34831) (Falcón-Pérez et al., 2005), and pGP-CMV-GCaMP6s was a gift from Dr. Douglas Kim (Addgene plasmid # 40753) (Chen et al., 2013). pCAG-PSD95.FingR-eGFP-CCR5TC (PSDFingR-GFP) was a gift from Dr. Don Arnold (Addgene plasmid # 46295) (Gross et al., 2013). The following constructs have been described before: Homer1c-mCherry (MacGillavry et al., 2013), GFP-Rab5, GFP-Rab7, mRFP-TfR (Hoogenraad et al., 2010), and tdTomato-Rab11 (Esteves da Silva et al., 2015). FUGW was a gift from David Baltimore (Addgene plasmid # 14883) (Lois et al., 2002). FUGW-mirShank-GFP was generated by replacing GFP with the Shank triple-knockdown cassette from pSM155-mirShank-GFP. All constructs were verified by sequencing.

Lentiviral particles were generated by transfecting the transfer plasmid together with the packaging plasmids p.MDG2 (Addgene plasmid #12259) and psPAX2 (Addgene plasmid #12260) (gifts from Didier Trono) in HEK293T cells. The supernatant was collected two days after transfection and concentrated using tangential flow filtration (Amicon Ultra spin filters, Millipore #UFC910024).

Confocal Imaging

Confocal images were taken with a Zeiss LSM 700 confocal laser-scanning microscope with a Plan-Apochromat 63x NA 1.40 oil objective. Images consist of a z stack of 7-9 planes at 0.39 μm interval, and maximum intensity projections were generated for analysis and display. The pERK1/2 (Figures 4J and 4K) and anti-mGluR5 (Figure S4B and S4C) images were taken with an EC Plan-Neofluar 40x NA 1.30 oil objective and consist of a z stack of 9 planes at 0.67 μm interval to obtain maximum intensity projections of the entire neuron in the z axis.

Antibody Feeding Assay

DIV18 neurons were transfected with myc-mGluR5 and endosomal markers as indicated and were live-labeled at DIV21 with mouse anti-c-*myc* (9E10, Santa Cruz Biotechnology, catalog # sc-40) diluted 1:200 in extracellular imaging buffer (120 mM NaCl, 3 mM KCl, 2 mM $CaCl_2$, 2 mM $MgCl_2$, 10 mM glucose, and 10 mM HEPES, pH adjusted to 7.35 with NaOH) for 30 minutes at RT, washed twice with imaging buffer, and incubated with 50 μM DHPG (Tocris) for the indicated time-points at 37°C. Cells were then fixed in 4% (w/v) paraformaldehyde (PFA) and 4% (w/v) sucrose in PBS for 10 minutes at RT, and washed three times with PBS supplemented with 100 mM glycine (PBS/Gly). To label the surface-expressed pool of receptors, cells were incubated with goat anti-mouse Alexa-647 (Thermo Fisher Scientific) diluted 1:200 in 5% (v/v) NGS in PBS/Gly for 30 minutes at RT, and washed three times with PBS/Gly. Then, to label the intracellular pool of receptors, cells were permeabilized with 0.25% (v/v) Triton X-100 and 5% (v/v) NGS in PBS/Gly for 5 minutes at RT, blocked with 10% (v/v) NGS in PBS/Gly for 30 minutes, and incubated with goat anti-mouse Alexa 488 (Thermo Fisher Scientific) diluted 1:200 in 5% (v/v) NGS in PBS/Gly for 30 minutes at RT. For co-labeling internalized mGluR5 with EEA1, cells were incubated with human anti-EEA1 (clone 4114; gift from M. Fritzler) diluted 1:500 in 5% (v/v) NGS in PBS/Gly for 2 hours at RT after the permeabilization and blocking steps, and detected with goat anti-human Alexa-568 (Thermo Fisher Scientific). Cells were washed three times with PBS/Gly, mounted in Mowiol mounting medium and imaged on a confocal system as described above.

For the Shank knockdown experiments DIV14 neurons were transfected with pSM155-Cer3 or mirShank::Cer3 together with myc-mGluR5 and Homer1c-mCherry. For the rescue experiments DIV14 neurons were transfected with indicated mirShank::mCherry-SHANK rescue constructs and myc-mGluR5. After 7 days (DIV21), neurons were live-labeled with anti-myc, stimulated with DHPG for 30 minutes, and the surface and internalized pools of myc-mGluR5 were visualized as described above.

For the density of internalized mGluR5 puncta in the dendritic shaft after treatment with DHPG for several points before fixation, DIV14 neurons were transfected with pSM155-mCherry or mirShank::mCherry and myc-mGluR5. After 7 days (DIV21), neurons were live-labeled with anti-myc, stimulated with DHPG for 0, 2, 5, 10 or 20 minutes, and the surface and internalized pools of myc-mGluR5 were visualized as described above.

Endocytic Zone Associated PSDs

For the Shank knockdown experiments DIV14 neurons were transfected with pSM155-Cer3 or mirShank::Cer3 together with GFP-CLC and Homer1c-mCherry. Alternatively, pSM155-Cer3 or mirShank::Cer3 transfected neurons were stained for endogenous clathrin with mouse anti-clathrin heavy chain (clone X22, Fisher Scientific) and Homer1, with rabbit anti-Homer1 (SySy), and visualized with goat anti-mouse Alexa-647 and goat anti-rabbit Alexa 488 antibodies. For the rescue experiments DIV14 neurons were

transfected with indicated mirShank::mCherry-SHANK rescue constructs and GFP-CLC. After 7 days (DIV21), neurons were fixed with 4% PFA and 4% sucrose in PBS for 15 minutes, washed, mounted in Mowiol mounting medium and imaged on a confocal system as described above.

For the co-localization between the PSD and Homer1c in control and Shank knockdown neurons, DIV14 neurons were transfected with pSM155-Cer3 or mirShank::Cer3 together with Homer1c-mCherry and PSDFingR-GFP. For the rescue experiments DIV14 neurons were transfected with indicated mirShank::mCherry-SHANK2 rescue constructs and PSDFingR-GFP. After 7 days (DIV21) the neurons were fixed, mounted and imaged as described above.

Receptor Recycling Assay

Neurons were live labeled with anti-myc 1:200 in extracellular imaging buffer for 30 minutes at RT, washed twice with imaging buffer, and incubated with 50 μM DHPG for 30 minutes at 37°C to induce receptor internalization. Remaining surface-bound anti-myc antibodies were blocked by incubating with HRP-conjugated swine anti-mouse (Agilent) antibodies diluted 1:100 for 30 minutes at RT. Cells were then washed twice and returned to 37°C to allow receptor recycling for the indicated time points. The recycled receptor pool was then labeled with goat anti-mouse Alexa-647 diluted 1:200 in 5% (v/v) NGS in PBS/Gly for 30 minutes at RT. Cells were washed three times with PBS/Gly, mounted in Mowiol mounting medium and imaged on a confocal system as described above.

Endogenous mGluR5 Protein Levels

DIV14 neurons were transfected with pSM155-mCherry or mirShank::mCherry and stained for endogenous surface and intracellular mGluR5 with rabbit anti-mGluR5 (Chemicon, catalog #ab5675) diluted 1:500 in 0.1% (v/v) Triton X-100 and 5% (v/v) NGS in PBS/Gly overnight at 4°C, and visualized with goat anti-rabbit Alexa 488 diluted 1:250 in 0.1% (v/v) Triton X-100 and 5% (v/v) NGS in PBS/Gly for 1 hour at RT. Cells were washed three times with PBS/Gly, mounted in Mowiol mounting medium and imaged on a confocal system as described above.

ERK1/2 Phosphorylation Assay

Neurons were transfected with pSM155-mCherry or mirShank::mCherry at DIV14. Tetrodotoxin (2 μM; TTX) was added 12 hours before treatment. At DIV22 neurons were incubated with either 100 μM DHPG diluted in extracellular imaging buffer for 10 or 30 minutes, or with extracellular imaging buffer only for non-treated control neurons. After the indicated time points the neurons were fixed in 4% PFA and 4% sucrose in PBS for 10 minutes at RT, followed by a quick wash with PBS/Gly, incubated with ice cold methanol (MeOH) for 10 minutes at −20°C and washed three times with PBS/Gly. The pSM155-mCherry or mirShank::mCherry transfected neurons were stained for ERK1/2 phosphorylation with rabbit anti-pERK1/2 (Cell Signaling, catalog #9101) diluted in 0.1% (v/v) Triton X-100 and 5% (v/v) NGS in PBS/Gly overnight at 4°C, and visualized with goat anti-rabbit Alexa-A488 diluted in 0.1% (v/v) Triton X-100 and 5% (v/v) NGS in PBS/Gly for 1 hour at RT. Cells were washed three times with PBS/Gly, mounted in Mowiol mounting medium and imaged on a confocal system as described above.

Live-Cell Imaging

Live-cell imaging was performed on a spinning disk confocal system (CSU-X1-A1; Yokogawa) mounted on a Nikon Eclipse Ti microscope (Nikon) with Plan Apo VC 100x 1.40 NA, or Plan Apo 60x 1.30 NA oil objectives (Nikon) with excitation from Cobolt Calyspso (491 nm), and Jive (561 nm) lasers, and emission filters (Chroma). The microscope was equipped with a motorized XYZ stage (ASI; MS-2000), Perfect Focus System (Nikon), Evolve 512 EM-CCD camera (Photometrics), and was controlled by MetaMorph 7.7.6 software (Molecular Devices). Neurons were maintained in a closed incubation chamber (Tokai hit: INUBG2E-ZILCS) at 37°C in 5% CO_2 in extracellular imaging buffer.

Live-Cell Imaging of SEP-Tagged mGluR5

DIV14 neurons were transfected with SEP-mGluR5 or SEP-mGluR1 together with pSM155-mCherry, mirShank::mCherry, mirShank::mCherry-Shank1 rescue, mirShank::mCherry-SHANK2 rescue, mirShank::mCherry- SHANK3 rescue or mCherry-Shank2 overexpression constructs. After 7 days, live neurons were imaged on a spinning disk confocal system (described above). After a 2-minute base-line acquisition, internalization was induced by the addition of DHPG to a final concentration of 50 μM and the SEP-mGluR5 signal was imaged every 30 s for 30 minutes (61 frames) using the 491 nm excitation laser. Dynasore (100 μM; Tocris) was added 2 minutes before acquisition. In the vehicle control extracellular imaging buffer was added to the incubation chamber after a 2-minute base-line acquisition in the same volume (40 μl to 360 μl) as DHPG. To control for photobleaching the SEP-mGluR5 signal was imaged every 5 minutes for 30 minutes (7 frames). Multiple Z stacks (10 planes) were obtained, with 0.5 μm intervals to acquire 4.5 μm image stacks.

SEP pH Sensitivity Assay

DIV18 neurons were transfected with SEP-mGluR5 and imaged at DIV21 on a spinning disk confocal system (described above). First, neurons were maintained in extracellular imaging buffer with pH 7.35 to visualize the mGluR5 surface pool. Then, the buffer was exchanged for imaging buffer with pH 5.5 (identical to extracellular imaging buffer, except 10 mM HEPES was replaced by 15 mM MES). Then, the low-pH buffer was exchanged for a buffer with pH 7.35 containing ammonium chloride (NH_4Cl) (identical

to extracellular imaging buffer, except for 70 mM NaCl, 50 mM NH_4Cl and 2 mM $NaHCO_3$ instead of 120 mM NaCl). To evaluate the change in fluorescence upon exchanging the buffers, each neuron was imaged consecutively for all three conditions and 6 time points at 30 s intervals were obtained per condition. Multiple Z stacks (10 planes) were obtained, with 0.5 μm intervals to acquire 4.5 μm image stacks per time point. For analysis, MAX intensity projections were used to assess the SEP-mGluR5 intensity for all 6 time points per condition and the change in fluorescence over time and different conditions was plotted.

Live-Cell Imaging of AcidiFluor ORANGE Halo-Tagged mGluR5

DIV18 neurons were transfected with Halo-mGluR5 and psm155-GFP, and imaged at DIV21 on a spinning disk confocal system (described above). Surface Halo-mGluR5 was labeled with 1.5 μM HaloTag AcidiFluor ORANGE (Goryo Chemical, cat#-GC310) for 20 minutes at 37°C in 5% CO_2. Neurons were rinsed in extracellular imaging buffer to remove unbound dye. Halo-mGluR5 labeled with AcidiFluor ORANGE was imaged in extracellular imaging buffer at 100 ms exposure and 2 s interval for 5 minutes using the 561 excitation laser. Timelapses were taken of a single z-plane. After a 40 s baseline acquisition, internalization was induced by the application of DHPG to a final concentration of 100 μM. Then, after 280 s, imaging buffer was exchanged for a buffer with pH 7.35 containing NH_4Cl (described above) to quench the signal of internalized Halo-mGluR5 AcidiFluor ORANGE. Also, a Z stack (10 planes) was obtained, with 0.5 μm intervals to acquire 4.5 μm image stacks of psm155-GFP, which was co-transfected for quantification purposes.

Fluorescence Recovery after Photobleaching

For fluorescence recovery after photobleaching (FRAP) experiments, DIV14 neurons were transfected with Venus-mGluR5 and pSM155-mCherry or mirShank::mCherry, and imaged on a spinning disk confocal system (described above). FRAP experiments were performed using the ILas2 system (Roper Scientific). Individual spines were photobleached with a targeted 491 nm laser and imaged every 5 seconds for fluorescence recovery for a period of 5 minutes.

Calcium Imaging

DIV14 neurons were transfected with GCaMP6s together with pSM155-mCherry or mirShank::mCherry and imaged 5 – 7 days later. Calcium imaging was performed on a spinning disk confocal system (described above). GCaMP6s signal was imaged at 2 s intervals (0.5 Hz) with a z stack stream (3 - 5 planes) at every time point. After 5 minutes baseline imaging, DHPG was added to 100 μM final concentration, and cells were imaged for another 5 - 10 minutes. MPEP (5 μM; Tocris) was added 5 minutes after application of DHPG.

Western Blot and Imaging

DIV10 neurons were infected with FUGW or FUGW-mirShank lentivirus for 10 days. Neurons were directly lysed in SDS sample buffer containing DTT. Lysates were subjected to Tris-Glycine SDS-PAGE followed by transfer on PVDF membranes. Blots were blocked in 2% BSA in PBS-T (0.05% Tween20) followed by primary and IRDye-conjugated secondary antibody incubation (in 2% BSA in PBS-T). Western blots were scanned using Odyssey infrared imaging system (Li-COR Biosciences).

QUANTIFICATION AND STATISTICAL ANALYSIS

Quantification of Endocytic Zone Associated PSDs

To quantify the fraction of synapses with an associated endocytic zone, circular regions with a fixed diameter (0.69 μm) were centered on the Homer1c-mCherry or mCherry-SHANK2 clusters to indicate synaptic regions. These regions were then transferred to the GFP-CLC or anti-clathrin channel. A synapse was classified EZ-positive if the clathrin cluster overlapped partially or completely with the circular region. The fraction of EZ-positive synapses was calculated per neuron and averaged per condition over the total population of neurons. Furthermore, the density of clathrin puncta was determined along the dendrite (per 10 μm).

To quantify the percentage of PSDFingR-GFP puncta overlapping with indicated mCherry constructs, puncta were selected with circular regions in the mCherry channel and transferred to the PSDFingR-GFP channel. It was classified as overlapping if the PSDFingR cluster overlapped partially or completely with the circular region. Furthermore, the puncta density of the indicated mirShank::mCherry-SHANK2 rescue constructs was determined along the dendrite (per 20 μm).

Quantification of Internalized mGluR5 Puncta in Spines and Dendrites

The number of PSDs associated with an internalized mGluR5 puncta was determined similar as the fraction of endocytic zone positive PSDs.

The density of internalized mGluR5 puncta in the dendritic shaft was determined by semi-automatic quantification. The dendritic shaft (20 μm in length) was selected and a threshold was set for each image. The selection was converted to an inverted binary image and a particle analysis was used to detect internalized mGluR5 puncta with a minimum size of 0.01 μm^2. The baseline condition (t = 0) was similar between control and Shank knockdown neurons. Therefore, to show the relative increase in internalized mGuR5 puncta in the dendritic shaft over time the treatment conditions were normalized per batch to the average density of its corresponding baseline condition.

Quantification of SEP-mGluR5 Internalization in Spines and Dendrites

MAX intensity projections of the Z stacks were obtained and corrected for XY drift over time using the Fiji plugin “StackReg.” To quantify the SEP-mGluR5 intensity over a time-period of 30 minutes circular regions of interest (spines or dendrites) were selected at $t = -2$ and the intensity was measured for all 61 time points. To obtain the change in relative fluorescence intensity ($\Delta F/F_0$) over time, background was subtracted and the intensity relative to $t = -2$ was computed. For visualization all values were subtracted by 1 and plotted at 1 minute intervals.

Quantification of AcidiFluor ORANGE Halo-Tagged mGluR5 Acidification in Spines and Dendrites

MAX intensity projections of the psm155-GFP Z stacks were made and used to trace the neuron using Fiji software. This selection was then transferred to the AcidiFluor ORANGE Halo-mGluR5 channel to clearly indicate the outline of the neuron. A Gaussian blur (sigma = 2) was applied to the AcidiFluor ORANGE images, and a total of 6 neurons from 2 batches were manually screened for acidification events. To test the pH sensitivity of AcidiFluor ORANGE, imaging buffer was exchanged for a buffer containing NH_4Cl which quenched the signal. Representative images are shown at 4-10 s intervals. To visualize the change in relative fluorescence intensity over time, values were plotted as $\Delta F/F_0$ for the spine and dendrite.

Quantification of Spine Enrichment

To assess the spine enrichment of surface mGluR5, the SEP-mGluR5 intensity at $t = -2$ min from the live-cell base-line acquisition was quantified in control, mirShank and mirShank::Shank1, mirShank::SHANK2 and mirShank::SHANK3 rescue neurons as relative spine intensity over relative dendritic shaft intensity. For each neuron circular regions were traced on multiple dendritic spines to measure spine intensity and for each selected spine a circular region in the dendrite at the base of the spine was measured as dendritic shaft intensity. Background intensity was subtracted.

Quantification of Immunofluorescence of Endogenous mGluR5

For the analysis of endogenous total mGluR5 levels a dendritic stretch of 20 μm was selected and traced in the mCherry channel using Fiji software. This selection was then transferred to the anti-mGluR5 channel and the average intensity of the anti-mGluR5 fluorescence of the transfected neurons with indicated constructs was obtained. Per batch the average intensity was normalized to the average intensity of the control neurons.

Quantification of FRAP Experiment

For FRAP analysis, the mean intensity of the bleached area was corrected for background values, as well as the bleaching that occurred during image acquisition. Data were normalized to control fluorescence averaged over 5 frames before bleaching. Individual recovery curves were fitted with a single-exponential function $I = A(1 - exp(-Kt))$ to estimate the mobile fraction (*A*) and time constant tau.

Quantification of Calcium Experiment

For each neuron, the fluorescence intensity of GCaMP6s signal was measured in 10 - 20 ROIs along the dendrite, background subtracted and averaged. To obtain the mean amplitude and frequency of the calcium oscillations, events were detected with the MATLAB ‘PeakFinder’ function.

Quantification of ERK1/2 Phosphorylation Assay

For analysis the cell bodies were manually traced based on the mCherry channel using Fiji software. The average intensity of the anti-pERK1/2 fluorescence of the transfected neurons with indicated constructs was obtained for each condition and was normalized per batch to the average intensity of the non-treated control condition.

Statistical Analysis

Statistical significance was tested with a paired t test (Figures 3L and 4G) or unpaired t test (Figures 3C, 3H, 4D, S3A, and S4A, S4D, and S4F) when comparing two groups with a normal distribution and a Mann Whitney test when comparing two groups without a normal distribution (Figures 4J and S3C). If multiple groups were compared (Figures 1D, 3D, 3F, 3J, S3G, S3H, S3J, and S4B), statistical significance was tested with a one-way ANOVA followed by a Tukey’s multiple comparison when comparing the mean of each column to the mean of every other column or a Dunnet’s multiple comparison when comparing the mean of each column to the mean of a control column. When comparing multiple groups without a normal distribution, a Kruskall Wallis followed by a Dunn’s multiple comparison was performed (Figure S3F). To test for an effect of treatment over time between different groups with matched values in time (Figures 1G–1L, 2B–2F, S1B, S1C, S1I– S1L, S2C, and S2D), statistical significance was tested with a repeated-measures two-way ANOVA followed by a Tukey’s multiple comparison when comparing more than two groups. To test for an effect of treatment over time between different groups without matched factors (Figures 4B and 4L), statistical significance was tested with a two-way ANOVA followed by a Tukey’s multiple comparison when comparing more than two groups. The data table of Figure 2G contains some missing values, since during image acquisition some frames were out of focus and could not be taken into account for analysis, and a mixed effects ANOVA was performed. The effect was considered significant if the row factor (time or treatment), the column

factor (condition) and the interaction (time x condition) effect were all significant (*P*-value below 0.05). In the text the *P*-values of the condition effects are reported. In the figures, * indicates significance based on the condition effect and when comparing more than two groups, * indicates significance based on the multiple comparison test. In all figures * was used to indicate a *P*-value < 0.05, ** for $p < 0.01$, and *** for $p < 0.001$. See Table S2 for all *P*-values and statistical tests performed. Data are represented as mean ± SEM. Reported *n* is number of neurons, which are indicated as scatters in the bar graphs. Each experiment was replicated in cultures from at least 2 independent preparations. Statistical analysis and graphs were prepared in GraphPad Prism and figures were generated in Adobe Illustrator CC.

DATA AND CODE AVAILABILITY

The published article includes all datasets generated or analyzed during this study.

Cell Reports, Volume *29*

Supplemental Information

Shank Proteins Couple the Endocytic Zone to the Postsynaptic Density to Control Trafficking and Signaling of Metabotropic Glutamate Receptor 5

Nicky Scheefhals, Lisa A.E. Catsburg, Margriet L. Westerveld, Thomas A. Blanpied, Casper C. Hoogenraad, and Harold D. MacGillavry

Supplementary Figure 1

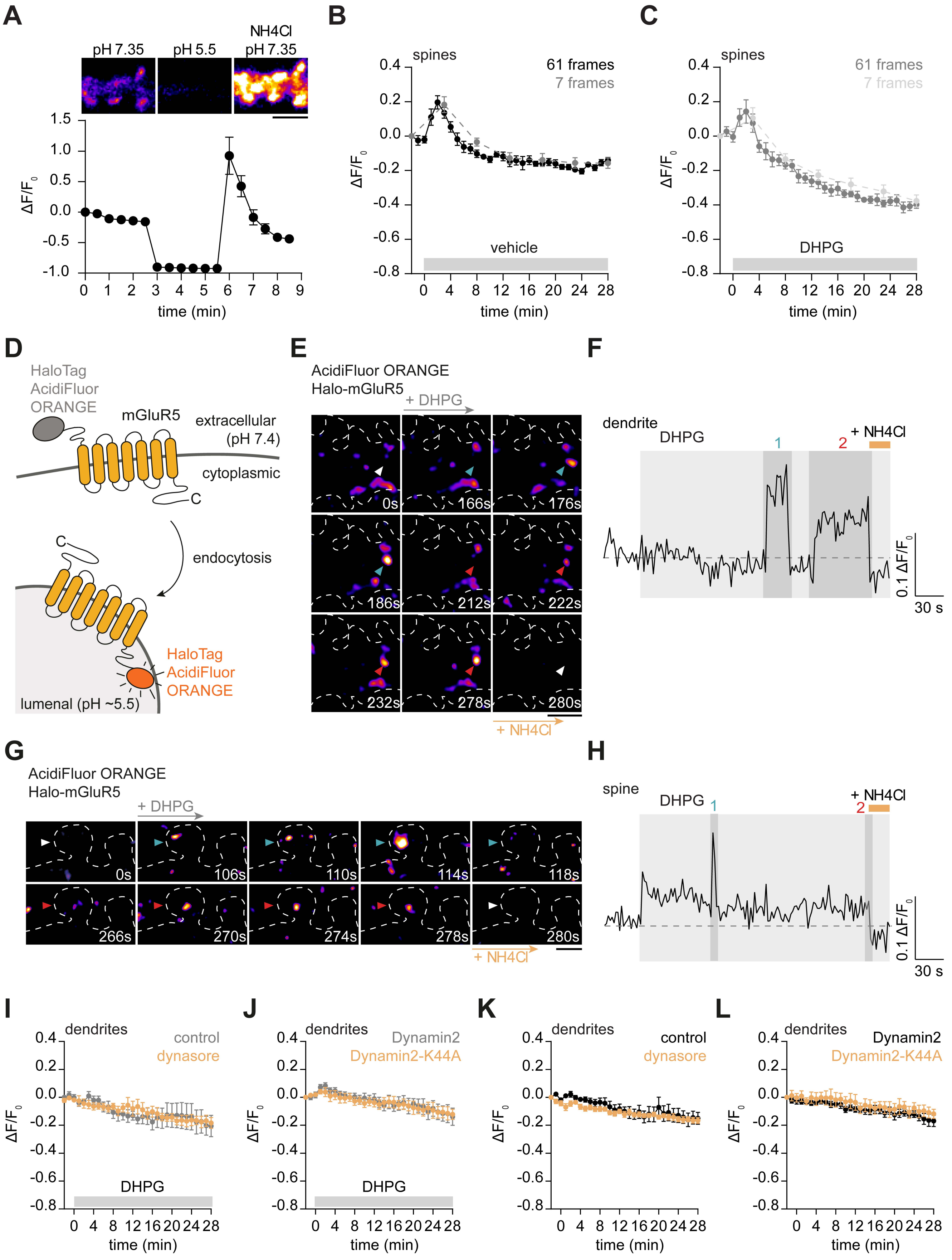

Figure S1, related to Figure 1. Characterization of SEP-mGluR5 fluorescence and evaluation of dendritic internalization

(A) Surface SEP-mGluR5 fluorescence intensity in imaging buffer with pH 7.35 (t = 0 - 3 min), is quenched at pH 5.5 (t = 3 – 6 min) and increased in fluorescence upon the application of imaging buffer containing NH_4Cl with pH 7.35 (t = 6 min) visualizing both surface and intracellular SEP-mGluR5 (n = 6). Scale bar, 2 µm.

(B and C) Quantification of SEP-mGluR5 intensity in spines over a 30-minute time period comparing the loss of SEP-mGluR5 intensity when imaged every 5 minutes (7 frames; dashed light grey line; n = 12) and when imaged every 30 seconds (61 frames; solid black line; n = 8) after application with vehicle (B) and after DHPG stimulation (7 frames; dashed light grey line; n = 12 and 61 frames; solid dark grey line; n= 6) (C). The data sets from 61 frames shown in B and C are also shown in Figure 1G, as these figures describe different aspects of the same experiment.

(D) Schematic of Halo-tag labeled with AcidiFluor ORANGE fused to mGluR5 to reveal acidification of Halo-mGluR5-containing endocytic vesicles.

(E and G) Representative image of a dendrite expressing Halo-mGluR5 labeled with AcidiFluor ORANGE stimulated with DHPG (at t = 40 s) showing two acidification events, and quenching of the signal upon application of imaging buffer containing NH_4Cl with pH 7.35 (t = 280 s) at the dendritic shaft (E) and in a spine (G). Arrowheads indicate two acidification events (1; blue and 2; red). Scale bars, 2 µm.

(F and H) $\Delta F/F_0$ trace of the Halo-mGluR5 signal intensity, showing the baseline (t = 0 – 40 s), application of DHPG (t = 42 – 178 s; light grey), the acidification events shown in E and G (dark grey) and the application of NH_4Cl (t = 280 – 300 s; orange) at the dendritic shaft shown in E (indicated by arrowhead) (F) and in the spine shown in G (indicated by arrowhead) (H).

(I and J) Quantification of SEP-mGluR5 intensity in dendrites over time after DHPG stimulation comparing the time course of SEP-mGluR5 intensity in control neurons (grey; n = 6) with neurons pre-treated with dynasore (orange; n = 6) (I) and in neurons co-transfected with Dyn2 (grey; n = 6) with neurons co-transfected with the dominant negative Dyn2-K44A (orange; n = 6) (J).

(K and L) Quantification of SEP-mGluR5 intensity in dendrites over time without the addition of DHPG comparing the time course of SEP-mGluR5 intensity in control neurons (black; n = 6) with neurons pre-treated with dynasore (orange; n = 8) (K) and in neurons co-transfected with Dyn2 (grey; n = 6) with neurons co-transfected with the dominant negative Dyn2-K44A (orange; n = 6) (L).

Data are represented as mean ± SEM.

Supplementary Figure 2

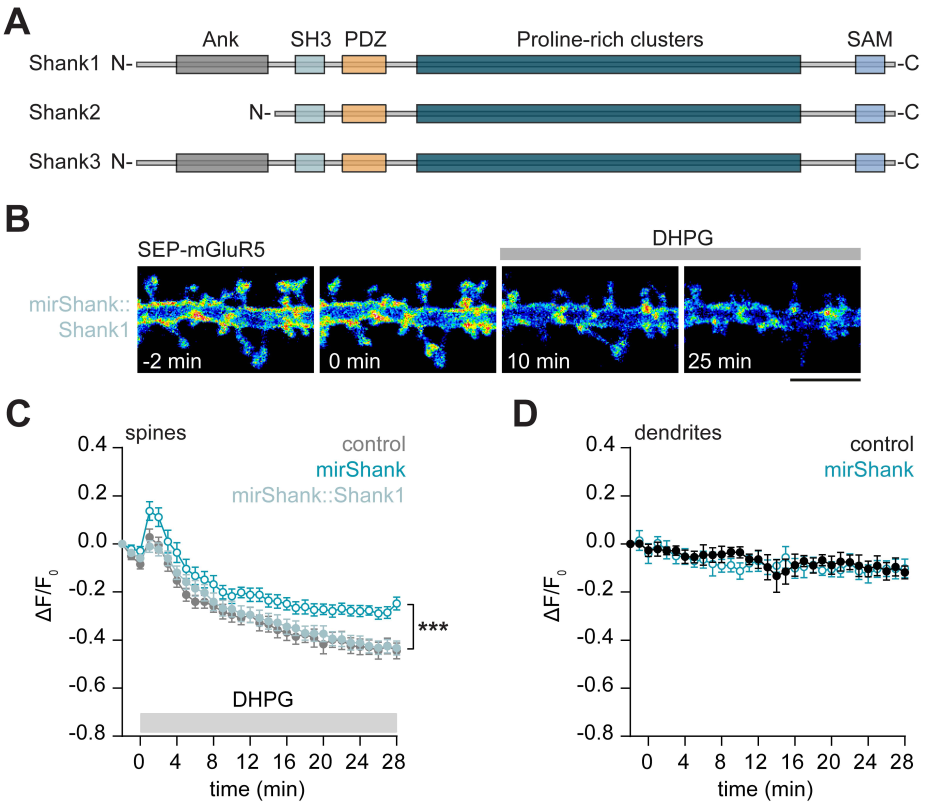

Figure S2, related to Figure 2. Re-expression of Shank1 rescues agonist-induced mGluR5 internalization in spines

(A) Domain structure of Shank1, Shank2 and Shank3.

(B) Representative live-cell time-lapse image of SEP-mGluR5 before and after DHPG stimulation (added at t = 0 min) in mirShank::Shank1 neurons. Scale bar, 5 µm.

(C) Quantification of SEP-mGluR5 intensity in spines over time after the addition of DHPG comparing the time course of SEP-mGluR5 intensity in control (grey; n = 14), mirShank (blue; open circles; n = 17) and mirShank::Shank1 rescue neurons (shade of blue; closed circles; n = 22).

(D) Quantification of SEP-mGluR5 intensity in dendrites over time without the addition of DHPG comparing the time course of SEP-mGluR5 intensity in control (black; n = 5) and mirShank neurons (blue; n = 5).

Data are represented as mean ± SEM. ***, indicates $P < 0.001$.

Supplementary Figure 3

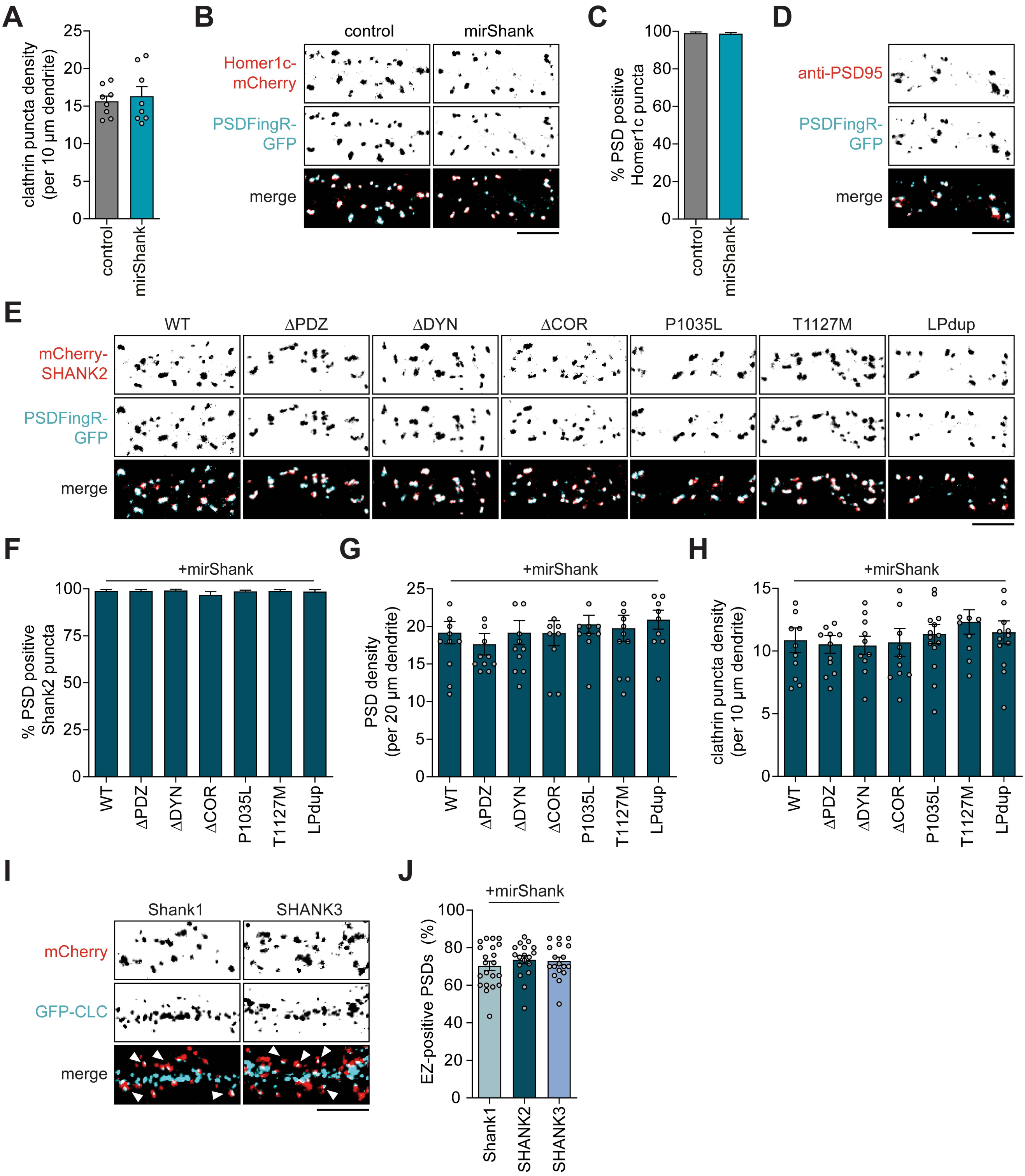

Figure S3, related to Figure 3. Clathrin puncta density in dendrites, mGluR5 spine enrichment and synaptic targeting of SHANK2 mutants

(A) Quantification of the density of GFP-CLC puncta along the dendrite (per 10 µm) in control (n = 8) and mirShank (n=8) neurons.

(B) Representative images of dendrites co-expressing PSDFingR-GFP (cyan) and Homer1c-mCherry (red) in control and mirShank neurons. Scale bar, 5 µm.

(C) Quantification of the percentage of Homer1c-mCherry puncta positive for PSDFingR-GFP, a marker of the PSD, in control (n = 10) and mirShank (n = 11) neurons.

(D) Co-localization of PSDFingR-GFP (cyan) and immuno-labeled anti-PSD-95 (red). Scale bar, 5 µm.

(E) Representative images of dendrites co-expressing mCherry-tagged SHANK2 rescue constructs (red) and PSDFingR-GFP (cyan). Scale bar, 5 µm.

(F) Quantification of the percentage of mCherry-tagged WT and mutant SHANK2 puncta positive for PSDFingR-GFP, a marker of the PSD (WT: n = 12, Δ PDZ: n = 11, ΔDYN: n = 12, ΔCOR: n = 10, P1035L: n = 11, T1127M: n = 12, LPdup: n = 10).

(G) Quantification of the density of PSDs, marked by PSDFingR-GFP, along the dendrite (per 20 µm) in neurons co-expressing the mCherry-tagged SHANK2 rescue constructs (WT: n = 12, Δ PDZ: n = 11, ΔDYN: n = 12, ΔCOR: n = 10, P1035L: n = 11, T1127M: n = 12, LPdup: n = 10).

(H) Quantification of the density of GFP-CLC puncta along the dendrite (per 10 µm) in neurons co-expressing mCherry-tagged SHANK2 rescue constructs (WT: n = 11, Δ PDZ: n = 12, ΔDYN: n = 10, ΔCOR: n = 11, P1035L: n = 15, T1127M: n = 10, LPdup: n = 13).

(I) Representative images of dendrites co-expressing mCherry-tagged Shank1, SHANK2 and SHANK3 rescue constructs (red) and GFP-CLC (cyan). Scale bar, 5 µm.

(J) Quantification of the percentage of EZ-positive PSDs in neurons co-expressing mCherry-tagged Shank1 (n = 21), SHANK2 (n = 18) and SHANK3 (n = 18) rescue constructs.

Data are represented as mean ± SEM.

Supplementary Figure 4

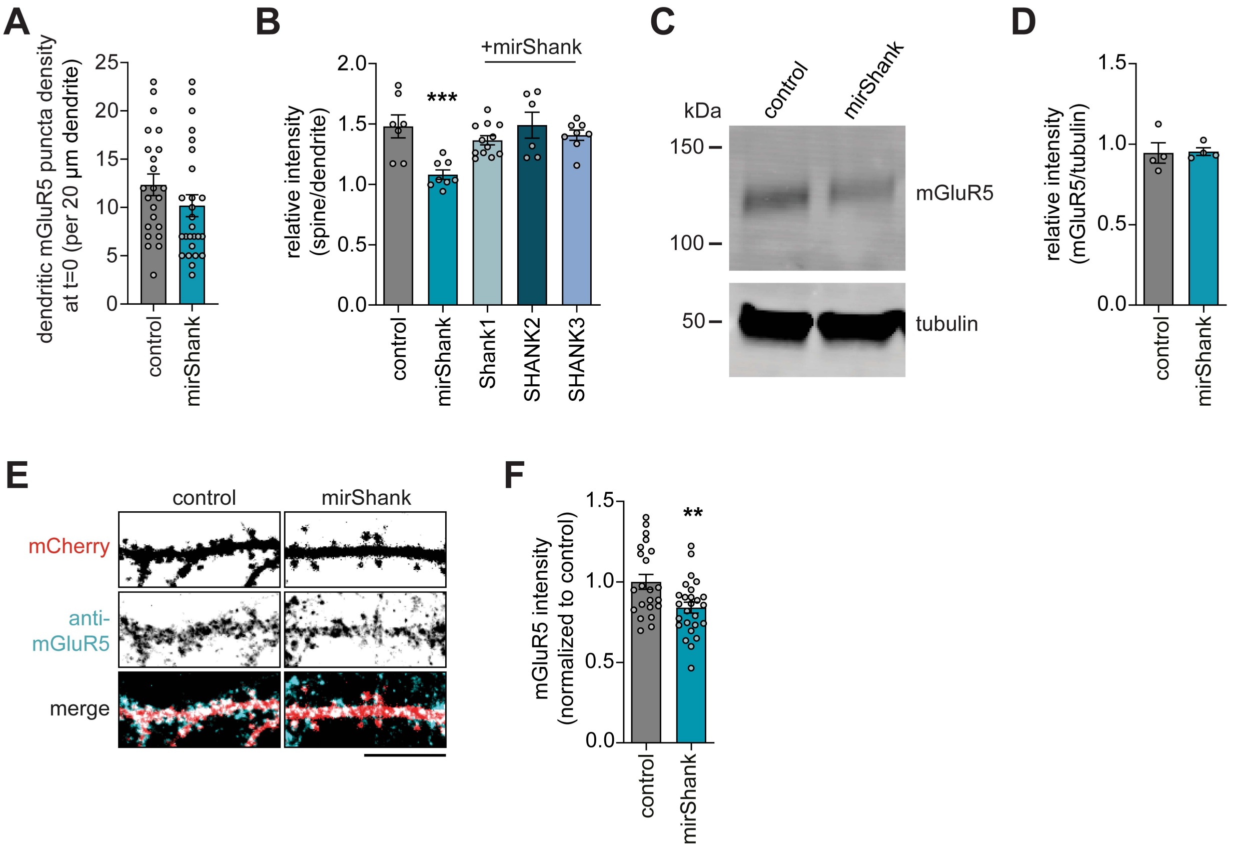

Figure S4, related to Figure 4. Shank proteins control surface and total expression of mGluR5

(A) Quantification of internalized myc-mGluR5 puncta density in dendrites of control (n = 23) and mirShank neurons (n = 26) at t = 0.
(B) Quantification of SEP-mGluR5 intensity in spines over dendritic shaft of control (n = 7), mirShank (n = 8), mirShank::Shank1 (n = 12), mirShank::SHANK2 (n = 6) and mirShank::SHANK3 (n = 9) rescue neurons.
(C) Western blot analysis of total lysates of neurons infected with GFP and GFP::mirShank and immuno-labelled for anti-mGluR5; tubulin was used as a loading control.
(D) Quantification of anti-mGluR5 over tubulin intensity in control and mirShank neurons (n = 3).
(E) Representative images of dendrites immuno-labeled for anti-mGluR5 (cyan) in mCherry-tagged control and mirShank neurons (red). Scale bar, 10 µm.
(F) Quantification of anti-mGluR5 intensity along the dendrite (20 µm) in control (n = 20) and mirShank (n = 27) neurons, normalized to the average intensity of anti-mGluR5 fluorescence in the control condition.
Data are represented as mean ± SEM. **, indicates $P < 0.01$ and ***, indicates $P < 0.001$.

Table S1, related to STAR Methods. miRNA targeting sequences

Targeting protein	miRNA sequence	Source
Shank1	ACAGACCAACCTGGATGAGAA	(MacGillavry et al., 2015)
Shank2	GGACTTGGATGAGGACTTTCT	(MacGillavry et al., 2015)
Shank3	GGAAGTCACCAGAGGACAAGA	(MacGillavry et al., 2015)

Table S2, related to STAR Methods. Statistical Analysis

Figure	Test	Condition	*P* value condition		***P value condition x time***
**1D**	ANOVA	ALL	<0.0001	***	
	ANOVA with TUKEY	EEA1 vs. Rab5	0.3715	ns	
		EEA1 vs. Rab11	0.9063	ns	
		EEA1 vs. TfR	0.8299	ns	
		EEA1 vs. Rab7	<0.0001	***	
		EEA1 vs. LAMP1	<0.0001	***	
		Rab5 vs. Rab11	0.9599	ns	
		Rab5 vs. TfR	0.9723	ns	
		Rab5 vs. Rab7	<0.0001	***	
		Rab5 vs. LAMP1	<0.0001	***	
		Rab11 vs. TfR	>0.9999	ns	
		Rab11 vs. Rab7	<0.0001	***	
		Rab11 vs. LAMP1	<0.0001	***	
		TfR vs. Rab7	<0.0001	***	
		TfR vs. LAMP1	<0.0001	***	
		Rab7 vs. LAMP1	0.6920	ns	
**1G**	2-way RM ANOVA	vehicle vs. DHPG	0.0008	***	<0.0001
**1H**	2-way RM ANOVA	vehicle vs. DHPG	0.1363	ns	0.1082
**1I**	2-way RM ANOVA	control vs. dynasore	0.0136	*	0.0001
**1J**	2-way RM ANOVA	control vs. Dyn2-K44A	0.0136	*	<0.0001
**1K**	2-way RM ANOVA	control vs. dynasore	0.6672	ns	<0.0001
**1L**	2-way RM ANOVA	control vs. Dyn2-K44A	0.9663	ns	0.9993
**S1B**	2-way RM ANOVA for 7 matching time points	61 frames vs. 7 frames	0.4483	ns	0.4409
**S1C**	2-way RM ANOVA for 7 matching time points	61 frames vs. 7 frames	0.3799	ns	0.9534
**S1I**	2-way RM ANOVA	control vs. dynasore	0.8814	ns	0.2119
**S1J**	2-way RM ANOVA	control vs. Dyn2-K44A	0.8080	ns	>0.9999
**S1K**	2-way RM ANOVA	control vs. dynasore	0.4029	ns	0.9983
**S1L**	2-way RM ANOVA	control vs. Dyn2-K44A	0.4755	ns	0.9829
**2B**	2-way RM ANOVA	control vs. mirShank	<0.0001	***	<0.0001
**2C**	2-way RM ANOVA	control vs. mirShank	0.5636	ns	0.0004
**2D**	2-way RM ANOVA	ALL	0.0026	**	<0.0001

	2-way RM ANOVA with Tukey	mirShank vs. Control<br>mirShank::SHANK2 vs. Control<br>mirShank::SHANK3 vs. Control<br>mirShank::SHANK2 vs. mirShank<br>mirShank::SHANK3 vs. mirShank<br>mirShank::SHANK3 vs. mirShank::SHANK2	<0.0001<br>0.9997<br>0.4561<br><0.0001<br><0.0001<br>0.5395	***<br>ns<br>ns<br>***<br>***<br>ns	
**2E**	2-way RM ANOVA	control vs. SHANK2 OE	0.4699	ns	<0.0001
**2F**	2-way RM ANOVA	control vs. mirShank	0.7244	ns	0.6593
**2G**	Mixed-effects ANOVA	control vs. mirShank	0.0001	***	<0.0001
**S2C**	2-way RM ANOVA	ALL	0.0082	**	<0.0001
	2-way RM ANOVA with Tukey	control vs. mirShank<br>control vs. mirShank::SHANK1<br>mirShank vs. mirShank::SHANK1	<0.0001<br>0.3235<br><0.0001	***<br>ns<br>***	
**S2D**	2-way RM ANOVA	control vs. mirShank	0.7976	ns	0.2373
**3C**	Unpaired t-test	control vs. mirShank	<0.0001	***	
**3D**	ANOVA	ALL	<0.0001	***	
	ANOVA with Tukey	untransfected vs. control<br>untransfected vs. mirShank<br>control vs. mirShank	0.5145<br>0.0001<br><0.0001	ns<br>***<br>***	
**3F**	ANOVA	ALL	<0.0001	***	
	ANOVA with Dunnet's	WT vs. ΔPDZ<br>WT vs. ΔDYN<br>WT vs. ΔCOR<br>WT vs. P1035L<br>WT vs. T1127M<br>WT vs. LPdup	0.9998<br><0.0001<br><0.0001<br><0.0001<br><0.0001<br>0.9979	ns<br>***<br>***<br>***<br>***<br>ns	
**3H**	Unpaired t-test	control vs. mirShank	0.0012	**	
**3J**	ANOVA	ALL	<0.0001	***	
	ANOVA with Dunnet's	WT vs. ΔPDZ<br>WT vs. ΔDYN<br>WT vs. ΔCOR<br>WT vs. P1035L<br>WT vs. T1127M<br>WT vs. LPdup	0.9999<br>0.0005<br>0.0068<br>0.0021<br>0.0003<br>0.8160	ns<br>***<br>**<br>**<br>***<br>ns	
**3L**	Paired t-test	30': EZ+ vs. EZ-<br>60': EZ+ vs. EZ-	0.0453<br>0.0123	*<br>*	
**S3A**	Unpaired t-test	control vs. mirShank	0.6554	ns	
**S3C**	Mann Whitney test	control vs. mirShank	0.8216	ns	
**S3F**	Kruskall Wallis	ALL	0.8980	ns	
	Kruskall Wallis with Dunn's	WT vs. ΔPDZ<br>WT vs. ΔDYN<br>WT vs. ΔCOR<br>WT vs. P1035L<br>WT vs. T1127M<br>WT vs. LPdup	>0.9999<br>>0.9999<br>>0.9999<br>>0.9999<br>>0.9999<br>>0.9999	ns<br>ns<br>ns<br>ns<br>ns<br>ns	
**S3G**	ANOVA	ALL	0.8440	ns	
	ANOVA with Dunnet's	WT vs. ΔPDZ	0.9523	ns	

		WT vs. ΔDYN	>0.9999	ns	
		WT vs. ΔCOR	>0.9999	ns	
		WT vs. P1035L	0.9903	ns	
		WT vs. T1127M	0.9996	ns	
		WT vs. LPdup	0.9268	ns	
**S3H**	ANOVA	ALL	0.7983	ns	
	ANOVA with Dunnet's	WT vs. ΔPDZ	0.9996	ns	
		WT vs. ΔDYN	0.9995	ns	
		WT vs. ΔCOR	0.9998	ns	
		WT vs. P1035L	0.9963	ns	
		WT vs. T1127M	0.7478	ns	
		WT vs. LPdup	0.9913	ns	
**S3J**	ANOVA	ALL	0.5627	ns	
	ANOVA with Tukey	Shank1 vs. SHANK2	0.5679	ns	
		Shank1 vs. SHANK3	0.7157	ns	
		SHANK2 vs. SHANK3	0.9715	ns	
**4B**	2-way ANOVA	control vs. mirShank	0.2719	ns	0.0129
	2-way ANOVA with Tukey	0':control vs. 2':control	>0.9999	ns	
		0':control vs. 5':control	0.4864	ns	
		0':control vs. 10':control	0.9977	ns	
		0':control vs. 20':control	0.6553	ns	
		0':mirShank vs. 2':mirShank	>0.9999	ns	
		0':mirShank vs. 5':mirShank	0.9301	ns	
		0':mirShank vs. 10':mirShank	0.0002	***	
		0':mirShank vs. 20':mirShank	0.6414	ns	
		2':control vs. 2':mirShank	>0.9999	ns	
		5':control vs. 5':mirShank	0.9997	ns	
		10':control vs. 10':mirShank	0.0108	*	
		20':control vs. 20':mirShank	>0.9999	ns	
**4C**	2-way RM ANOVA	control vs. mirShank	0.8781	ns	>0.9999
**4D**	Unpaired t-test	control vs. mirShank	0.8567	ns	
**4G**	Paired t-test	DHPG vs. DHPG + MPEP	0.0023	**	
**4J**	Mann Whitney test	control vs. mirShank	0.0043	**	
**4L**	2-way ANOVA	ALL	0.0007	***	0.1260
	2-way ANOVA with Tukey	NT:control vs. NT:mirShank	0.9997	ns	
		NT:control vs. 10':control	0.2464	ns	
		NT:control vs. 10':mirShank	0.9998	ns	
		NT:control vs. 30':control	0.0885	ns	
		NT:control vs. 30':mirShank	0.9889	ns	
		NT:mirShank vs. 10':control	0.1676	ns	
		NT:mirShank vs. 10':mirShank	>0.9999	ns	
		NT:mirShank vs. 30':control	0.0579	ns	
		NT:mirShank vs. 30':mirShank	0.9998	ns	
		10':control vs. 10':mirShank	0.1217	ns	
		10':control vs. 30':control	0.9986	ns	
		10':control vs. 30':mirShank	0.0354	*	
		10':mirShank vs. 30':control	0.0334	*	
		10':mirShank vs. 30':mirShank	0.9992	ns	

		30':control vs. 30':mirShank	0.0064	**	
**S4A**	Unpaired t-test	control vs. mirShank	0.1874	ns	
**S4B**	ANOVA	ALL	0.0003	***	
	ANOVA with Tukey	control vs. mirShank	0.0009	***	
		control vs. Shank1	0.6477	ns	
		control vs. SHANK2	>0.9999	ns	
		control vs. SHANK3	0.9299	ns	
		mirShank vs. Shank1	0.0094	**	
		mirShank vs. SHANK2	0.0011	**	
		mirShank vs. SHANK3	0.0059	**	
		Shank1 vs. SHANK2	0.6121	ns	
		Shank1 vs. SHANK3	0.9838	ns	
		SHANK2 vs. SHANK3	0.9029	ns	
**S4D**	Unpaired t-test	control vs. mirShank	0.9032	ns	
**S4F**	Unpaired t-test	control vs. mirShank	0.0064	**	
